# A high-throughput stereo-imaging system for quantifying rape leaf traits during the seedling stage

**DOI:** 10.1186/s13007-017-0157-7

**Published:** 2017-01-31

**Authors:** Xiong Xiong, Lejun Yu, Wanneng Yang, Meng Liu, Ni Jiang, Di Wu, Guoxing Chen, Lizhong Xiong, Kede Liu, Qian Liu

**Affiliations:** 1 Britton Chance Center for Biomedical Photonics, Wuhan National Laboratory for Optoelectronics, Huazhong University of Science and Technology, 1037 Luoyu Rd., Wuhan, 430074 People’s Republic of China; 2 National Key Laboratory of Crop Genetic Improvement and National Center of Plant Gene Research, Huazhong Agricultural University, Wuhan, 430070 People’s Republic of China; 3 College of Engineering, Huazhong Agricultural University, Wuhan, 430070 People’s Republic of China; 4 MOA Key Laboratory of Crop Ecophysiology and Farming System in the Middle Reaches of the Yangtze River, Huazhong Agricultural University, Wuhan, 430070 People’s Republic of China

**Keywords:** Stereo-imaging system, Canopy three-dimensional structure, Individual leaf traits, Morphological classification

## Abstract

**Background:**

The fitness of the rape leaf is closely related to its biomass and photosynthesis. The study of leaf traits is significant for improving rape leaf production and optimizing crop management. Canopy structure and individual leaf traits are the major indicators of quality during the rape seedling stage. Differences in canopy structure reflect the influence of environmental factors such as water, sunlight and nutrient supply. The traits of individual rape leaves traits indicate the growth period of the rape as well as its canopy shape.

**Results:**

We established a high-throughput stereo-imaging system for the reconstruction of the three-dimensional canopy structure of rape seedlings from which leaf area and plant height can be extracted. To evaluate the measurement accuracy of leaf area and plant height, 66 rape seedlings were randomly selected for automatic and destructive measurements. Compared with the manual measurements, the mean absolute percentage error of automatic leaf area and plant height measurements was 3.68 and 6.18%, respectively, and the squares of the correlation coefficients (R^2^) were 0.984 and 0.845, respectively. Compared with the two-dimensional projective imaging method, the leaf area extracted using stereo-imaging was more accurate. In addition, a semi-automatic image analysis pipeline was developed to extract 19 individual leaf shape traits, including 11 scale-invariant traits, 3 inner cavity related traits, and 5 margin-related traits, from the images acquired by the stereo-imaging system. We used these quantified traits to classify rapes according to three different leaf shapes: mosaic-leaf, semi-mosaic-leaf, and round-leaf. Based on testing of 801 seedling rape samples, we found that the leave-one-out cross validation classification accuracy was 94.4, 95.6, and 94.8% for stepwise discriminant analysis, the support vector machine method and the random forest method, respectively.

**Conclusions:**

In this study, a nondestructive and high-throughput stereo-imaging system was developed to quantify canopy three-dimensional structure and individual leaf shape traits with improved accuracy, with implications for rape phenotyping, functional genomics, and breeding.

**Electronic supplementary material:**

The online version of this article (doi:10.1186/s13007-017-0157-7) contains supplementary material, which is available to authorized users.

## Background

Oilseed rape (*Brassica napus*) is an important species that is cultivated in many countries for its valuable oil and protein [1–5]. The area planted with oilseed rape has rapidly increased in recent decades [6]. The leaf is of fundamental importance to the rape, acting as the power generator and aerial environmental sensor of the plant [7, 8]. Leaves are primarily involved in photosynthesis and transpiration, thereby influencing crop yield [9, 10]. The size, shape, area and number of leaves are of great significance to plant science, allowing scientists to distinguish between different species and even to model climate change [11]. Moreover, plant canopy architecture is of major interest for plant phenotyping. Variations in canopy structure have been linked to canopy function and have been attributed to genetic variability as well as a reaction to environmental factors such as light, water, and nutrient supplies as well as stress [12]. Thus, canopy structure is an essential variable for plant’s adaptation to its environment [13, 14]. It is therefore important to study the oilseed rape phenotypic traits of both individual leaf shape and plant canopy structure.

Many researchers have carried out studies of individual leaf traits [15–19]. In many cases, rapeseed species can be distinguished by aspects of leaf shape, flower shape, or branching structure. Shape is, of course, important in many other disciplines [11]. To characterize these properties, O’Neal et al. [20] applied a desk-top scanner and public domain software to extract individual leaf shape traits, including leaf height, leaf width. However, the efficiency of this process is problematic: each leaf must be removed from the plant and scanned into a digital format. In addition, some complex traits such as leaf serration and leaf margin can’t be assessed by this method. In an attempt to measure leaf area easier and more accurate, the new software namely “Compu Eye, Leaf & Symptom Area” was developed by Bakr et al. [21], etc. The purpose of this software is to obtain the symptom area for each leaf. But, this method offers no method to quantify leaf serration and morphology traits. Thus, this software has some limitations in practical. Igathinathane et al. [22] designed software that uses the computer monitor as the working surface to trace leaf outline and determines leaf area, perimeter, length, and width. This software offers no method to quantify leaf serration and inner cavity-related traits. Also, this is a semi-automatic program and the interactive processes are complex and tedious. Weight et al. [23] reported the development of LeafAnalyser, which is an excellent tool to facilitate PCA analysis of leaf shape parameters. However, the leaf petiole region did not remove when analyzing the leaf traits and the software was not released as open source, negating the possibility of further development by the community. Bylesjö et al. [7] designed a tool to extract classical indicators of blade dimensions and leaf area, as well as measurements that indicate asymmetry in leaf shape and leaf serration traits. This software not only obtains object boundaries but also analyzes serration traits. However, it requires the user to analyze leaves in vitro and to correctly characterize the blade azimuth for subsequent image analysis, which limits the throughput of the analysis. Dengkui et al. [24] designed a tool to acquire plant growth information by abstracting the plant morphological characters, size and color of leaves, etc. The morphological operation has been used to remove petiole, which will influence the accuracy of leaf margin information extraction. But, the method offers no method to quantify leaf serration and inner cavity-related traits. Yang et al. [25] designed a device “HLS” for assessing leaf number, area, and shape. The device is efficient and can process multiple blades in parallel. However, all blades must be cut from the plant before insertion into the HLS device. Furthermore, the present equipment for extracting serrated blade edge traits is insufficient.

The work described above focuses on the morphological traits of individual leaves. However, three-dimensional canopy structure also plays an important role in sustaining plant function. The canopy structure contains useful information regarding developmental stage during the vegetation period as well as yield-forming parameters [26, 27]. Three-dimensional imaging methods can be broadly classified into two types: active and passive [14]. Commonly used active light projection technologies include laser scanning and structured light. Light detection and ranging (LIDAR) laser scanners have emerged as a powerful active sensing tool for direct three-dimensional measurement of plant height, canopy structure, plant growth, and shape responses [28]. The precision of laser scanner systems is very high, but the scanning time is very long, reducing the system’s throughput. In structure light systems, the Kinect Microsoft RGB-depth camera [29] is used as a depth camera to shine light onto the object scene. The light reflected from the scene is used to build the depth image by measuring the deformation of the spatially structured lighting pattern [30]. The system produces 640 × 480 pixels RGB-depth images coded with a 16-bits dynamic that are acquired at a rate of 30 frames per second [13]. The imaging speed of these systems is very high, nearly satisfying the demands of real-time measurement. However, the measurement accuracy exhibits low spatial resolution in comparison with a standard RGB camera. One problem with laser-based and structured light systems is that they do not work well with reflective objects, and it is often necessary to coat the surface with a non-reflective layer that can lead to the collection of unsatisfactory texture data [31]. In addition, the method used for volumetric reconstruction from multiple images has been proposed to be a passive three-dimensional imaging technology [32–34]. It works by obtaining multiple images from different fixed angles. Here, a rotated plate is used to achieve multi-angle imaging, which will result in time-consuming rotations. Moreover, this method requires a significant amount of post-processing. Also, binocular/multi-view stereo imaging approach is another major passive three-dimensional imaging technology [35, 36]. There are some applications of using binocular/multi-view stereo vision for plant sensing. For automatic robot or vehicle-mounted system, the binocular stereo system is a common component for obtaining distance depth information or field plant 3D structure [37–40]. Moreover, the binocular stereo is also used in small- to medium-sized plant canopies reconstruction. Ivanov et al. [41] applied film-based stereo photogrammetry to reconstruct the maize canopy, where the plant canopy geometrical structure was analyzed and different simulation procedures were carried out to analyze leaf position and orientation and leaf area distribution. Andersen et al. [42] designed simulated annealing (SA) binocular stereo match algorithm for young wheat plants and analyzed height and total leaf area for single wheat plant. Biskup et al. [43] designed a stereo vision system with two cameras to build 3D models of soybean canopy and analyzed the angle of inclination of the leaves. Also, for isolated leaf, Biskup established a stereoscopic imaging system, which quantifies surface growth of isolated leaf discs floating on nutrient solution in wells of microtiter plates [44]. Müller-Linow et al. [12] developed a software package, which provides tools for the quantification of leaf surface properties within natural canopies via 3-D reconstruction from binocular stereo images. Furthermore, the multi-view stereo 3D reconstruction for plant phenotyping is also widely used combining with SfM- and MVS-based photogrammetric method. Lou et al. [45] described an accurate multi-view stereo (MVS) 3D reconstruction method of plants using multi-view images, which takes both accuracy and efficiency into account. Several plants, including arabidopsis, wheat and maize, are used to evaluate the performance of reconstruction algorithm. Rose et al. [46] developed a multi-view stereo system to evaluate the potential measuring accuracy of a SfM- and MVS-based photogrammetric method for the task of organ-level tomato plant phenotyping. The leaf area, main stem height and convex hull of the complete tomato plant are analyzed. Miller et al. [47] applied a low-cost hand-held camera to accurately extract height, crown spread, crown depth, stem diameter and volume of small potted trees. The multi-view stereo-photogrammetry was used to generate 3D point clouds. From the literatures above, the binocular stereo is usually used in small-sized plant canopies reconstruction by using two top-view cameras and the multi-view stereo 3D reconstruction method is applied for organ-level plant 3D phenotyping.

In this study, we attempt to create a three-dimensional surface model of the rape canopy from images taken by double top-view cameras, and we estimate geometric attributes such as plant height and canopy leaf area. For RGB images collected using a stereo-imaging system, a novel image analysis pipeline for the accurate quantification seedling rape leaf traits was developed. We are thus able to perform leaf shape analysis, including contour signatures and shape features.

## Results and discussion

### Development of a stereo-imaging system

**Fig. 1:**
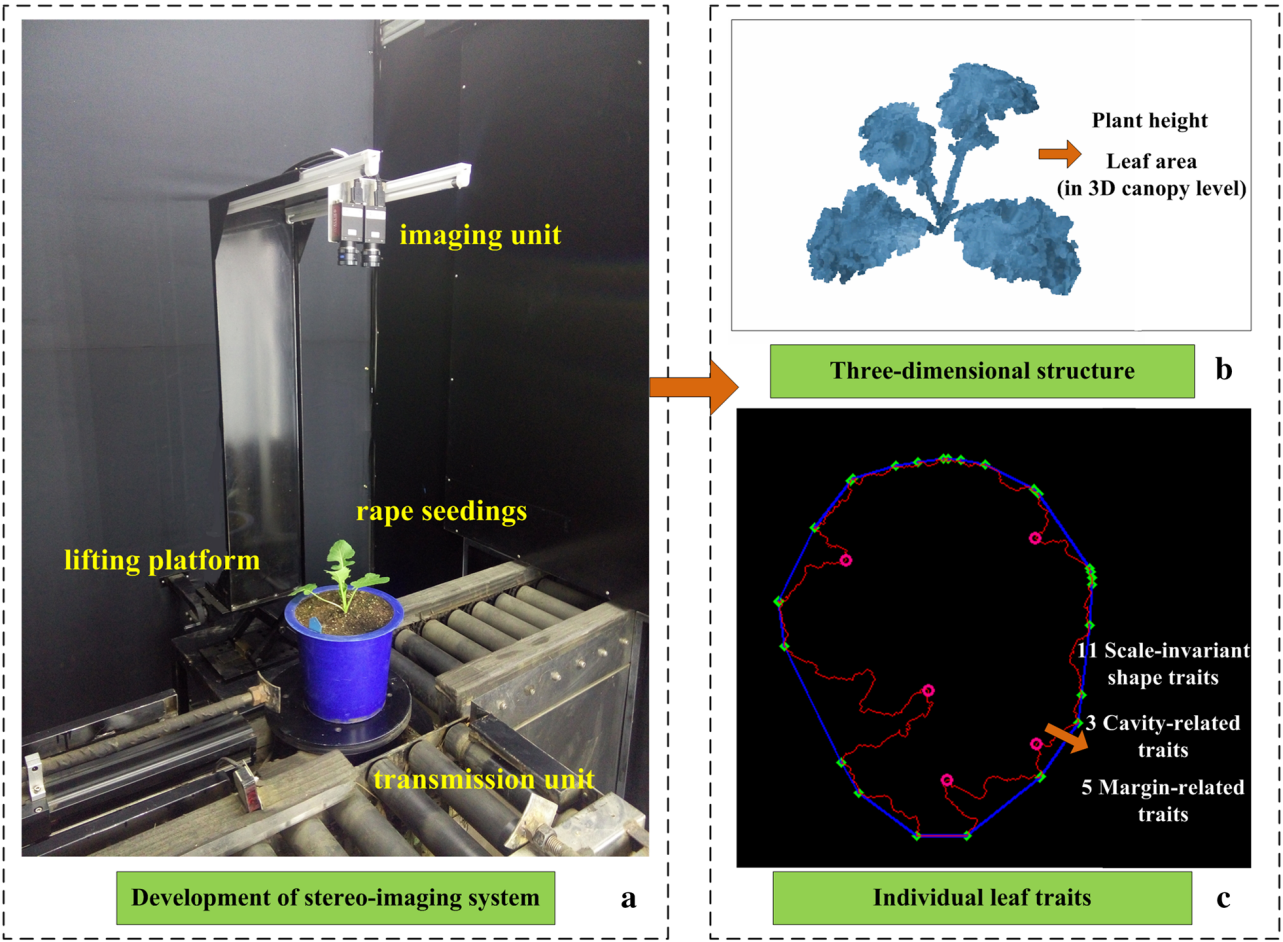
Development of stereo imaging system and two optional processing modes. **a** The inspection unit. **b** The three-dimensional structure of seedling rape canopy. We can extract plant height and canopy leaf area from the three-dimensional structure. **c** The individual rape leaf morphological traits, including scale-invariant shape traits, cavity-related traits and margin-related traits

In order to extract canopy leaf area, plant height and canopy three-dimensional structure, we developed a stereo-imaging system consisting of three major units: an imaging unit, a transportation unit and a control unit (Fig. 1a). For the imaging unit, we utilized two identical RGB cameras [AVT Stingray F-504B/F-504C, Allied Vision Technologies Corporation, 2452 (H) × 2056 (V) resolution] with 8 mm fixed focal lenses (M1214-MP, Computar Corporation), two LED lamps and a lifting platform. The RGB cameras are fixed to ensure that the two main optical axes are parallel and that the two imaging planes are located at the same horizontal level. An automatic trigger acquires image pairs, and software was developed to obtain the color image pairs simultaneously. The lifting platform can be used to adjust the imaging region [537.5 mm (H) × 449.9 mm (V)] and spatial resolution (0.2188 mm/pixel). In addition, the computer workstation (HP xw6400, Hewlett-Packard Development Company, USA) plays the role of central control unit, and utilizes software developed by LabVIEW 8.6 (Nation Instruments, USA) to communicate with the two RGB cameras. In order to achieve high-throughput measurements, the stereo-imaging system was integrated into an automated high-throughput phenotyping facility developed in our previous work [48]. Two optional processing modes were developed to reconstruct the three-dimensional structure of the seedling rape canopy (Fig. 1b) and to extract individual rape leaf traits (Fig. 1c).

### Three-dimensional structure of the seedling rape canopy

**Fig. 2:**
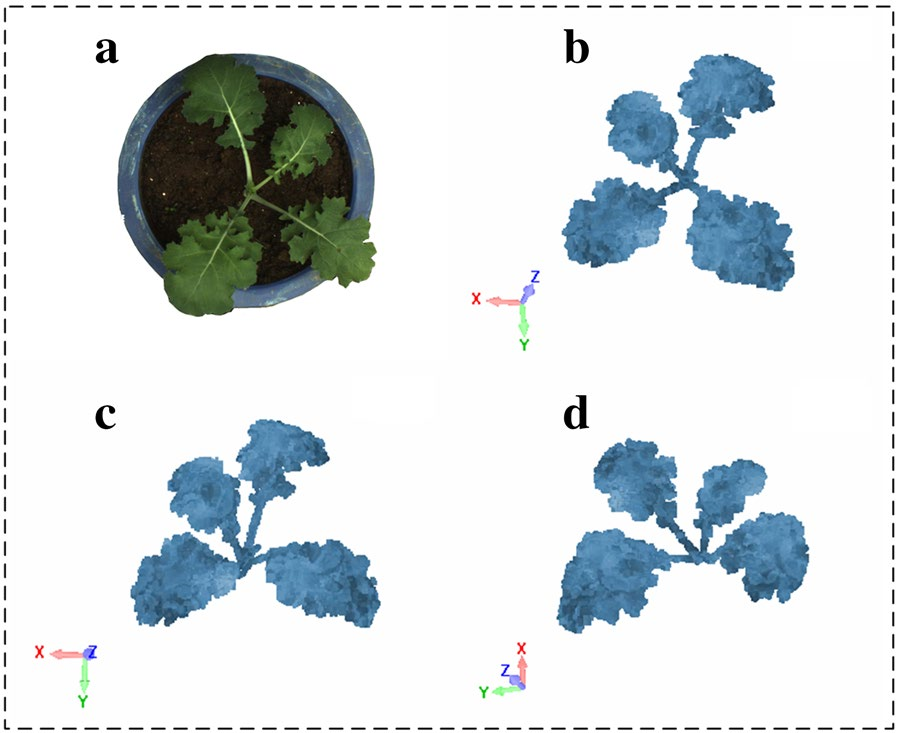
Three-dimensional reconstructions for seedling rape canopy at three different perspectives. **a** The original two-dimensional rape leaf image. **b**–**d** There are three types of perspectives for rape three-dimensional canopy structure

Plant canopy structure can be described by a range of complex and variable phenotypic traits that dictate the function of plant [49]. Here, canopy three–dimensional point cloud data were extracted from pairs of digital color images obtained under a constant light environment. The point cloud size for each canopy reconstruction is nearly 5.5 Mb. The user-friendly software interface for three-dimensional reconstruction is shown in Additional file 1: Figure S3, and the final reconstructed seedling rape canopy shown in Fig. 2 from three different perspectives.

### Leaf area and plant height

**Fig. 3:**
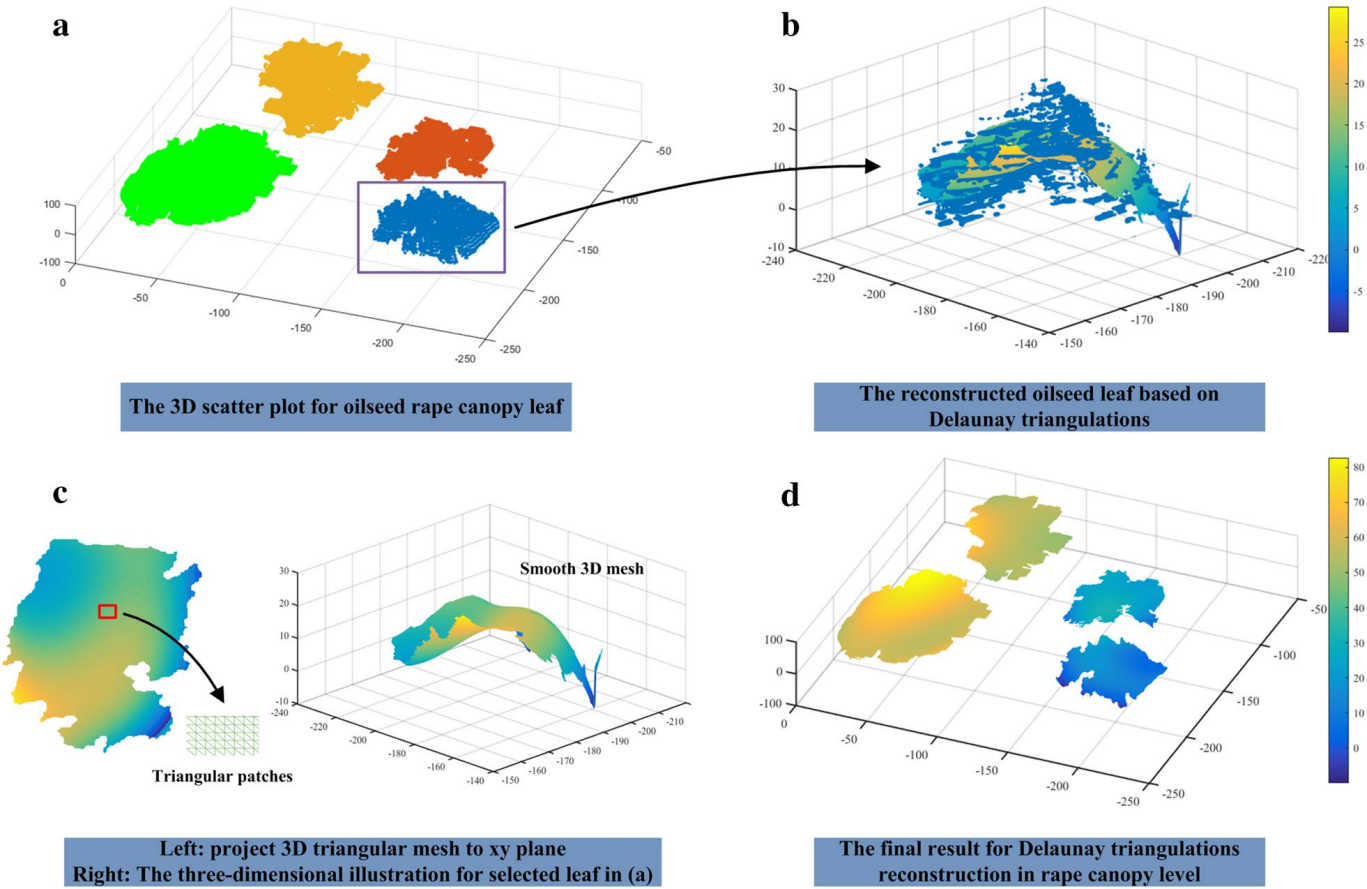
The schematic diagram for three-dimensional reconstruction based on Delaunay triangulations. **a** The three-dimensional scatter plot for oilseed rape canopy leaf (take an oilseed rape with four leaves, for example). *Color differences in point* cloud represents different rape leaf. **b** The reconstructed oilseed leaf [the rectangular area in image (**a**)] based on Delaunay triangulations. *Color differences* in smooth triangular mesh reflect the depth information in different leaf region. **c** The *left part* is the smooth 3D triangular mesh, which is projected to xy plane. The triangular patches image in the lower-right corner shows the local details in the red rectangle. The *right part* is the illustration for left oilseed rape leaf in three-dimensional space. **d** The final result for Delaunay triangulations reconstruction in canopy level. *Color differences* in smooth triangular mesh reflect the depth information in different leaf region

From the generated three-dimensional structure, we were able to extract two important parameters: leaf area and plant height. To evaluate leaf area based on canopy level, the Delaunay algorithm was used in the process of three-dimensional mesh generation. Figure 3 shows the detailed process for leaf triangular patches generation. After the stereo-imaging for seedling rape canopy, a set of 3D point cloud can be obtained (Fig. 3a). Color differences in point cloud represents different rape leaf. The matlab functions “trimesh” and “delaunay” based on Lifting Method [50] are applied to achieve Delaunay algorithm. The “delaunay” function produces an isolated triangulation, which is useful for applications like plotting surfaces via the “trimesh” function. The stack of triangular patches forms the 3D leaf region and a smoothing mechanism is used to extract smooth triangular mesh (Fig. 3b, c). Finally, we can obtain the Delaunay triangulations in rape canopy level and the sum area of all smooth triangular meshes is the canopy leaf area (Fig. 3d).

**Fig. 4:**
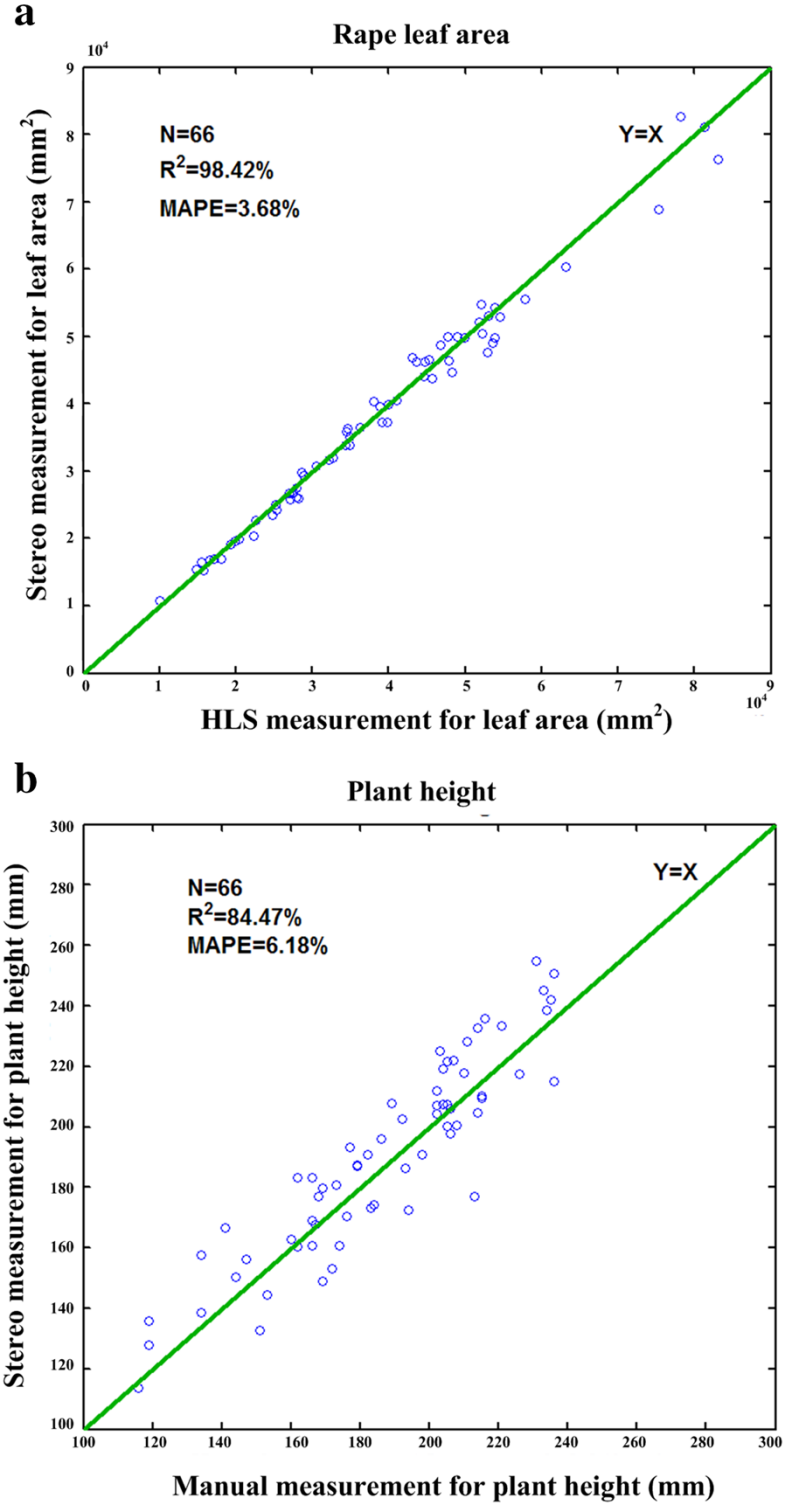
The performance evaluation of the leaf area and plant height. **a** The scatter plots of the stereo imaging measurement versus the HLS measurement for leaf area. **b** The scatter plots of the stereo imaging measurement versus the manual measurement by a ruler for calculating the plant height

To evaluate the measurement accuracy of leaf area and plant height (vertical distance from the edge of a plastic pot to the tip of longest leaf), 66 rape seedling plants were randomly selected for manual measurement. Figure 4 shows the results of manual observation versus automatic observation. The *MAPE* values were 3.68% for leaf area and 6.18% for plant height, and the squares of the correlation coefficients (*R* ^2^) were 0.984 and 0.845, respectively. Detailed experimental data are presented in Additional file 2. The result shows that the stereo-vision method has a good potential for accurate measurement.

### Individual leaf traits

**Fig. 5:**
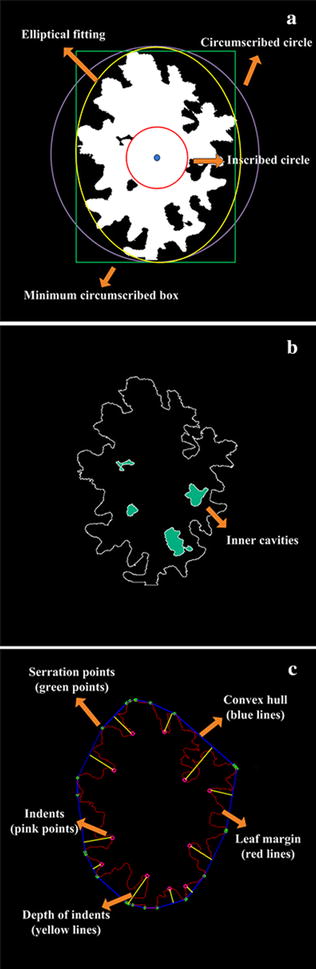
The extraction of individual leaf traits. **a** The extraction of the inscribed circle, circumscribed circle and minimum circumscribed box. **b** Analysis of the inner edges and cavities. **c** Calculate the leaf margin and indents by using the convex hull algorithm

Shape-based individual leaf traits, such as leaf size, vein network and leaf margin, are currently used for plant species identification and quantitative trait loci mapping [11, 16, 51, 52]. These shape-based morphological traits can be extracted using our image analysis pipeline. However, these traits alone do not reflect differences of leave shape due to variation in leaf size. We therefore must consider several new characteristic parameters that are not influenced by leaf size. Here, 19 shape related traits, including 11 scale-invariant traits, 3 inner cavity-related traits, and 5 margin-related traits, are proposed (Additional file 3). The definitions of all shape-related traits are shown in Fig. 5 and Table 1, and the computational formulas are provided in Eqs. 1–9.

**Table 1:** Definitions of nineteen leaf shape-related traits

Classification	Variable	Definition
Scale-invariant traits	AA	The aspect ratio of leaf minimum circumscribed box
	R	The ratio of leaf area to minimum circumscribed box area
	AC	The ratio of leaf area to leaf convex hull area
	PC	The ratio of leaf circumference to leaf convex hull perimeter
	S	The ratio of leaf area to the square of leaf convex hull perimeter
	E	The ratio of long axis of ellipse to short axis of ellipse
	FF	The ratio of leaf area to the square of leaf perimeter
	PAR	The ratio of leaf circumference to leaf area
	SFD	Reflect the effectiveness of the occupies space without image cropping [53]
	IFD	Reflect the effectiveness of the occupies space with image cropping [53]
	C	The ratio of inscribed circle radius to circumscribed circle radius
Cavity traits	NIC	The number of inner cavities
	APIC	The average perimeter of inner cavities
	AAIC	The average area of inner cavities
Margin related traits	TNI	Total number of indents
	ENI	Effective number of indents
	ADI	The average depth of indents
	ADEI	The average depth of effective indents
	AVE	The average calculated value of effectiveness

1 $$ Aspect\;Ratio = \frac{{Length_{bounding \, box} }}{{Width_{bounding \, box} }} $$

2 $$ Rectangularity = \frac{{Area_{object} }}{{Area_{bounding \, box} }} $$

3 $$ Area\;Convexity = \frac{{Area_{object} }}{{Area_{convex \, hull} }} $$

4 $$ Perimeter\;Convexity = \frac{{Perimeter_{object} }}{{Perimeter_{convex \, hull} }} $$

5 $$ Sphericity = \frac{4\pi \times {Area_{object} }}{{Perimeter_{convex \, hull}^{2} }} $$

6 $$ Eccentricity = \frac{{Axis\;Length_{long} }}{{Axis\;Length_{short} }} $$

7 $$ Form\;Factor = \frac{{4\pi \times Area_{object} }}{{Perimeter_{object}^{2} }} $$

8 $$ P/A = \frac{{Perimeter_{object} }}{{Area_{object} }} $$

9 $$ Circularity = \frac{{Radius_{inscribed \, circle} }}{{Radius_{excircle} }} $$

As seen in Fig. 5a, the red and purple circles represent the inscribed circle and circumscribed circle, respectively. The yellow ellipse indicates the result of elliptical fitting for a rape leaf. The green rectangle represents the minimum circumscribed box of the blade region. The inner cavities are by definition surrounded by a boundary region that is not connected to the outer boundary of the object. As seen in Fig. 5b, the green regions delineate inner cavities. In addition, the red lines (Fig. 5c), indicate the outer boundary of the individual leaf, while the blue lines demarcate the convex hull. The green points on the convex hull lines represent the serration points, which reflect to the vertices in all directions. The intermediate region between two serration points defines an indent. For each indent region, two serration points can be connected by a straight line, and the depth of the indent is measured as the longest distance from an indent point to the corresponding straight line. The effectiveness of the indents is calculated using the following Eq. 10. When the ratio is greater than 0.3, we consider the indents to be effective indents.

10 $$ iseffective = \frac{depth}{min (height,width)/2 } $$

where, *height* and *width* represent the number of minimum circumscribed box rows and cols, respectively. In addition, *depth* represents the distance from the indent point to the corresponding convex hull straight line.

The software interface for extracting individual leaf traits is shown in Additional file 4: Figure S4.

### Stepwise discriminant analysis

**Fig. 6:**
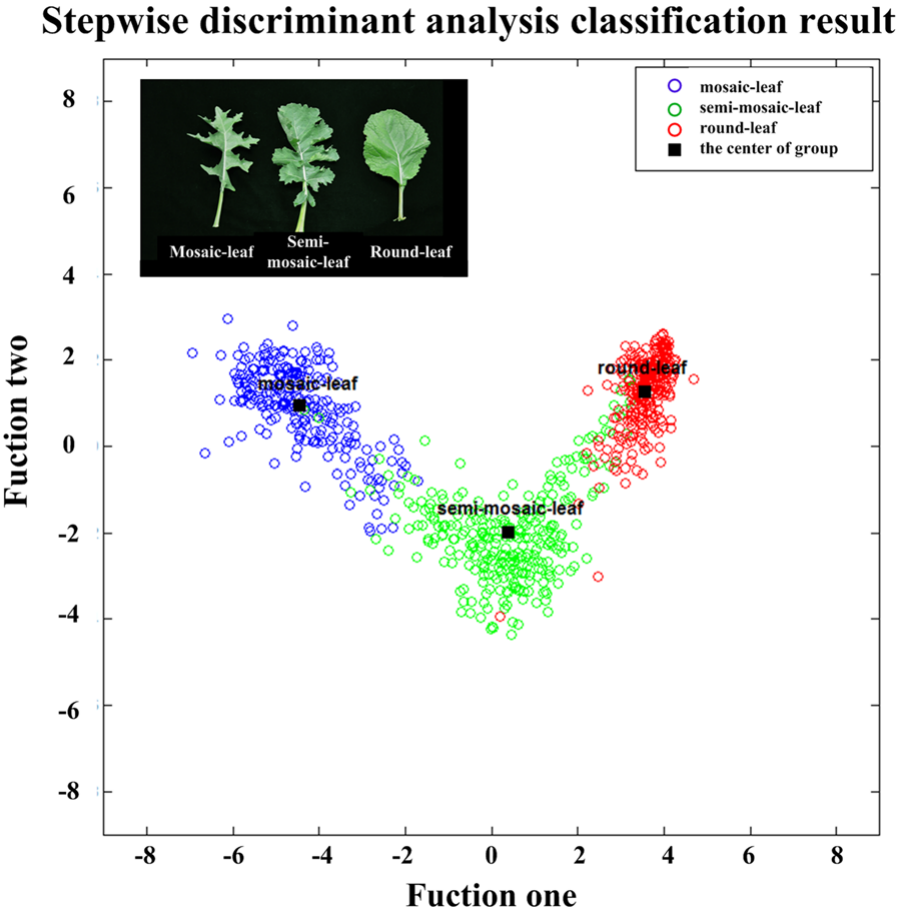
Stepwise discriminant analysis classification results. The abscissa and ordinate represent two classification functions, which were built by stepwise discriminant analysis algorithm. The *black squares* reflect the center of different groups, which can be calculated with two decision functions. The *red*, *green* and *blue points* represent round-leaf, semi-mosaic-leaf and mosaic-leaf, respectively

801 samples were randomly selected and divided into two groups: 402 samples with three different leaf shapes (mosaic-leaf, semi-mosaic-leaf, and round-leaf) comprising the training group and 399 samples with three different leaf shapes comprising the testing group. According to our classification, eleven of nineteen significant traits were selected by stepwise discriminant analysis as independent variables to construct two decision functions. The final classification using the two decision functions is shown in Fig. 6.

The black square blocks represent the center of the three different leaf shapes, which can be calculated using two decision functions. In Fig. 6 (red points mean round-leaf, green points mean semi-mosaic-leaf and blue points mean mosaic-leaf) and Table 2, the classification accuracy is given. We found that 92.7% of the tested grouped cases were correctly classified. In addition, we applied the leave-one-out cross-validation (LOO-CV) [54] to assess the accuracy of the classification model and found that 94.4% of the cross-validated grouped cases were correctly classified.

**Table 2:** Stepwise discriminant analysis classification results

	Leaf type	Predicted group membership			Total
		Mosaic	Semi-mosaic	Round	
Trained group	% Mosaic-leaf	98.3	1.7	0	100.0
	Semi-mosaic-leaf	1.4	90.3	8.3	100.0
	Round-leaf	0	0	100.0	100.0
Tested group	% Mosaic-leaf	97.5	2.5	0	100.0
	Semi-mosaic-leaf	7.6	88.9	3.5	100.0
	Round-leaf	0	7.4	92.6	100.0
Cross-validated^a^	% Mosaic-leaf	95.9	4.1	0	100.0
	Semi-mosaic-leaf	2.1	89.3	8.7	100.0
	Round-leaf	0	1.5	98.5	100.0

96.0% of trained grouped cases correctly classified

92.7% of tested grouped cases correctly classified

94.4% of cross-validated grouped cases correctly classified

^a^Cross validation is conducted only for those cases in the analysis. In cross validation, each case is classified by the functions derived from all cases other than that case

**Fig. 7:**
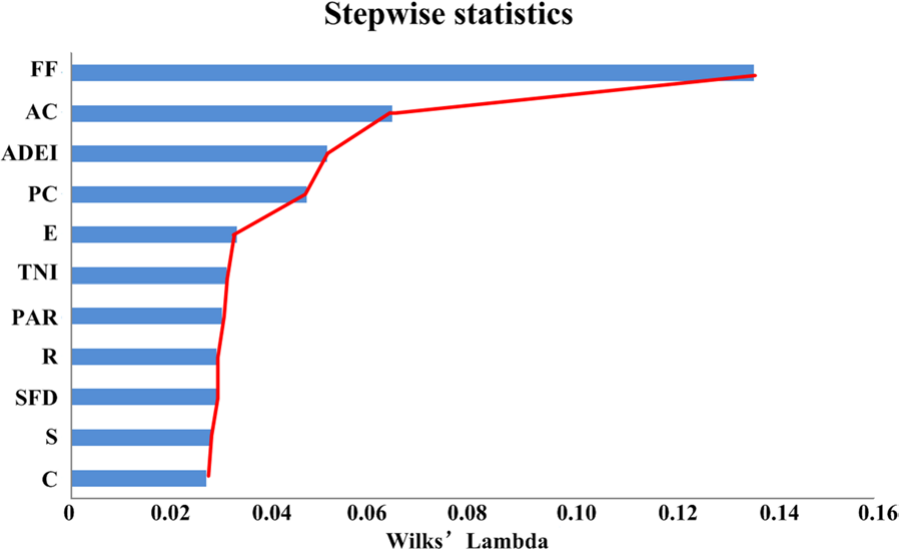
The performance analysis of traits in stepwise discriminant analysis. The screening results of the stepwise discriminant analysis. The traits from *top* to *bottom* reflect the order of characteristics screening. The value of Wilks’ Lambda statistic represents the discriminant ability after entering current traits

Stepwise discriminant analysis (SDA) [55] has been proven to effectively classify different rape leaf shapes. Moreover, in stepwise discriminant analysis, the first selected variable carries more weight in classification. In this study, the first four selected variables—form factor (FF), area convexity (AC), the average depth of effective indents (ADEI), and perimeter convexity (PC)—represent nearly 97% of the classification ability, as shown in Fig. 7. As we can see from Fig. 7, introducing a new variable will have little impact on the classification results after the selection of the first four variables. For FF, the round-leaf always has small perimeter for a given area, so the FF for the round-leaf shape is smaller than that of the mosaic-leaf and semi-mosaic-leaf. AC represents the ratio of the leaf area to the leaf convex hull area, which is an important parameter that reflects leaf morphology. For mosaic-leaf, the serrate border feature increases the convex area of the leaf. Thus, the duty ratio relative to its convex hull decreases markedly in comparison with the two other leaf shapes. Due to its serrated edge, the mosaic-leaf exhibits deeper indents compared with other two shapes.

### Support vector machine (SVM)

Support vector machine (SVM) is a standard classification technique that has been shown to produce state-of-the-art results in many classification problems [54, 56]. To apply SVM to our rape seedling leaf classification, nearly half of the 801 rape samples (402 samples) were used as training parameters and the other samples (399 samples) without labels were divided into a testing group for comparison of the results. We found that 94.7% of the tested grouped cases were correctly classified. In addition, the leave-one-out (LOO-CV) accuracy is 95.6% for the cross-validated group. The specific classification accuracy is shown in Table 3.

**Table 3:** SVM classification results

	Leaf type	Predicted group membership			Total
		Mosaic	Semi-mosaic	Round	
Trained group	% Mosaic-leaf	100.0	0	0	100.0
	Semi-mosaic-leaf	0	99.3	0.7	100.0
	Round-leaf	0	0	100.0	100.0
Tested group	% Mosaic-leaf	100.0	0	0	100.0
	Semi-mosaic-leaf	6.2	92.4	1.4	100.0
	Round-leaf	0	7.4	92.6	100.0
Cross-validated^a^	% Mosaic-leaf	96.7	3.3	0	100.0
	Semi-mosaic-leaf	3.1	93.1	3.8	100.0
	Round-leaf	0	2.6	97.4	100.0

99.8% of trained grouped cases correctly classified

94.7% of tested grouped cases correctly classified

95.6% of cross-validated grouped cases correctly classified

^a^Cross validation is conducted only for those cases in the analysis. In cross validation, each case is classified by the functions derived from all cases other than that case

### Random forest

In essence, the random forest (RF) model is a multiple decision trees classifier, and it is widely used in regression analysis and multi-classification [57, 58]. In this study, 402 samples with three different shapes were used to construct random forest model. The other 399 samples with category labels as testing group were applied to evaluate the performance of classification. The final classification accuracy for a test group is 91.7%, and the leave-one-out cross-validation accuracy is 94.8%. The specific classification accuracy is shown in Table 4.

**Table 4:** Random forest classification results

	Leaf type	Predicted group membership			Total
		Mosaic	Semi-mosaic	Round	
Trained group	% Mosaic-leaf	100.0	0	0	100.0
	Semi-mosaic-leaf	0	100.0	0	100.0
	Round-leaf	0	0	100.0	100.0
Tested group	% Mosaic-leaf	99.2	0.8	0	100.0
	Semi-mosaic-leaf	8.3	87.5	4.2	100.0
	Round-leaf	0	10.4	89.6	100.0
Cross-validated^a^	% Mosaic-leaf	96.7	3.3	0	100.0
	Semi-mosaic-leaf	3.5	91.0	5.5	100.0
	Round-leaf	0	3.0	97.0	100.0

100.0% of trained grouped cases correctly classified

91.7% of tested grouped cases correctly classified

94.8% of cross-validated grouped cases correctly classified

^a^Cross validation is conducted only for those cases in the analysis. In cross validation, each case is classified by the functions derived from all cases other than that case

### A comparison of the performance of the three methods of classification

As shown in Table 5, all three methods were able to satisfactorily classify leaves. The final classification accuracy for test group (399 samples) is 92.7, 94.7, 91.7% for stepwise discriminant analysis (SDA), support vector machine (SVM) and random forests (RF), respectively. In addition, the leave-one-out cross validation classification accuracy is 94.4% for SDA, 95.6% for SVM and 94.8% for RF algorithm. Among them, the stepwise discriminant analysis has a better prediction effect on round-leaf, while the support vector machine classifier is the most sensitive to mosaic-leaf. From the perspective of predicated group accuracy and the leave-one-out cross-validated results, the most reliable forecasting model was established by SVM algorithm.

**Table 5:** A comparison of three classification methods

Methods of classification	Predicted group accuracy				Leave-one-out cross-validated (LOO-CV)
	Mosaic	Semi-mosaic	Round	Total (n = 399)	
Stepwise discriminant analysis	98.3%	90.3%	100.0%	92.7%	94.4%
Support vector machine (SVM)	100%	92.4%	92.6%	94.7%	95.6%
Random forests classifier	99.2%	87.5%	89.6%	91.7%	94.8%

Fixed half selected for training (402), and the other half (399) for testing

### The performance of efficiency and accuracy

In this work, the stereo-imaging system is integrated to the high-throughput phenotyping facility. Each pot-grown rape would be transported by the conveyor, and the image pairs were acquired by the two top-view cameras at the same time. The inspection procedure is fully automated and highly efficient (45 s per plant) [48]. All the image processing works are carried out after the completion of image acquiring. Here, the time for image processing consists of two parts: canopy 3D reconstruction and individual leaf traits extraction. Usually, the time for canopy three-dimensional reconstruction is closely linked to the size of oilseed rape. After evaluated with 10 different size seedling rape samples, the average processing time for each canopy 3D reconstruction and data extraction of leaf area and plant height is about 43.46 s; for manual interaction, the time for each individual leaf extraction is about 8 s. The detailed description for manual part is shown in video (Additional file 5). Moreover, the two independent parts could run in parallel to save time. In this way, the total processing time depends on the longer part. So, the manual interaction does not lag the efficiency of the high-throughput platform. In addition, the manual interaction method can extract individual leaf with more accuracy compared with automatic segmentation, and the efficiency is also satisfactory. Assuming that the system can work 8 h a day, then, about 660 pots can handle just one day, which is an acceptable number for high-throughput.

**Fig. 8:**
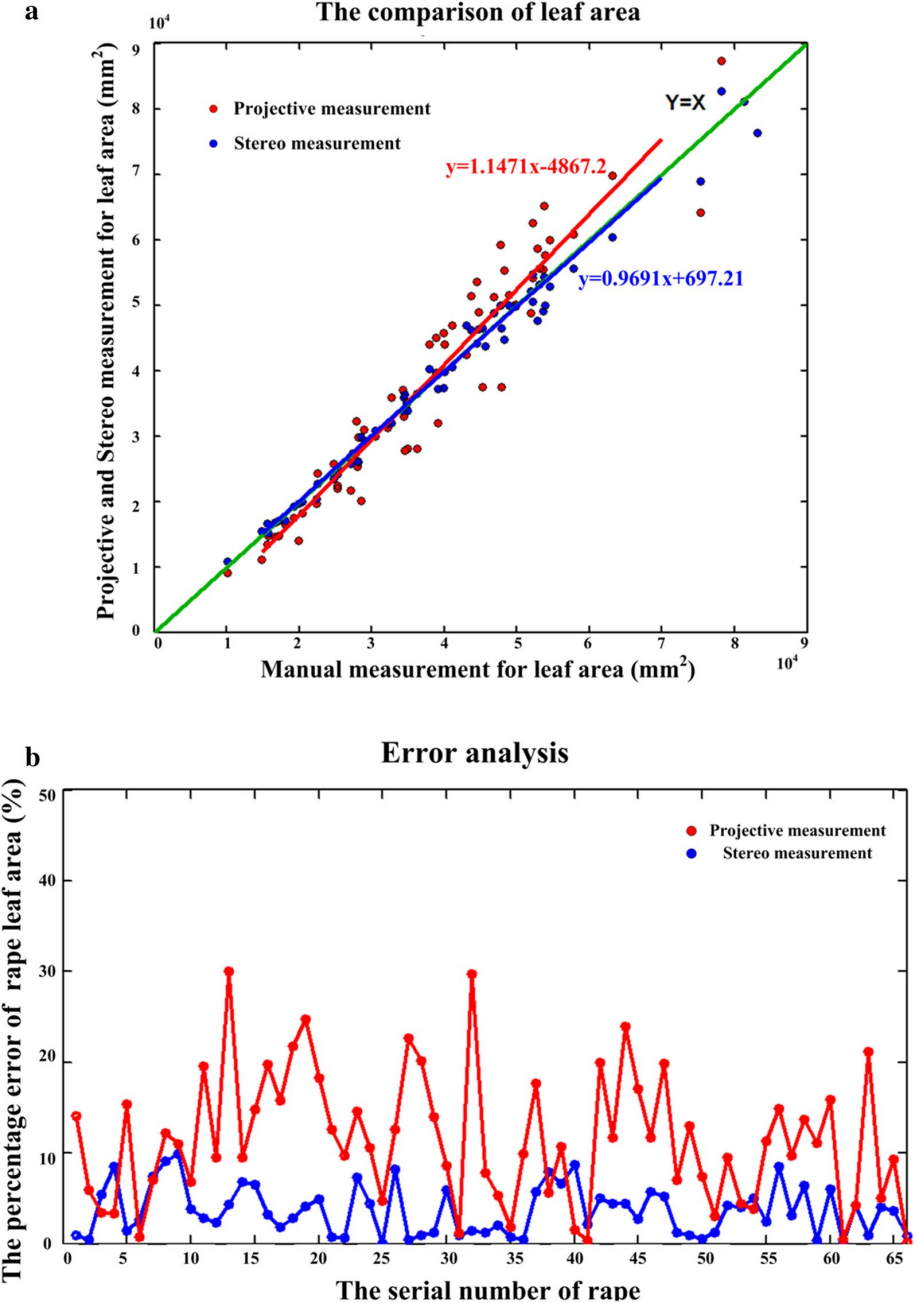
The comparison of two different methods for leaf area. **a** The *red scatter points* represent the leaf area result of the two-dimensional projective method by only using one top image. The *blue scatter points* indicate the result of three-dimensional measurement by using stereo imaging algorithm for leaf area. **b** The distribution of percentage error with two different methods

From the view of measurement accuracy for rape seedling leaf area, a comparison result of two different methods is shown in Fig. 8a. The red scatter points represent the leaf area result of two-dimensional projective method by only use one top-view image. After the rape was segmented from the background in the top-view image, the area of each rape is calculated by multiplying the pixel area and average spatial resolution, while blue scatter points indicate the result of three-dimensional stereo measurement by analyzing canopy structure. The *MAPE* values were 3.68% for three-dimensional stereo measurement and 11.44% for two-dimensional projective measurement, and the square of correlation coefficients (*R* ^2^) for three- and two-dimensional measurements was 0.984 and 0.938, respectively. Obviously, compared with two-dimensional imaging, the stereo measurement considering more spatial information of rape leaf can indicate more accuracy of leaf area in real world. Moreover, the area errors are almost below eight percent by using the stereo measurement method (Fig. 8b).

### The overlapping situation in stereo-imaging

**Fig. 9:**
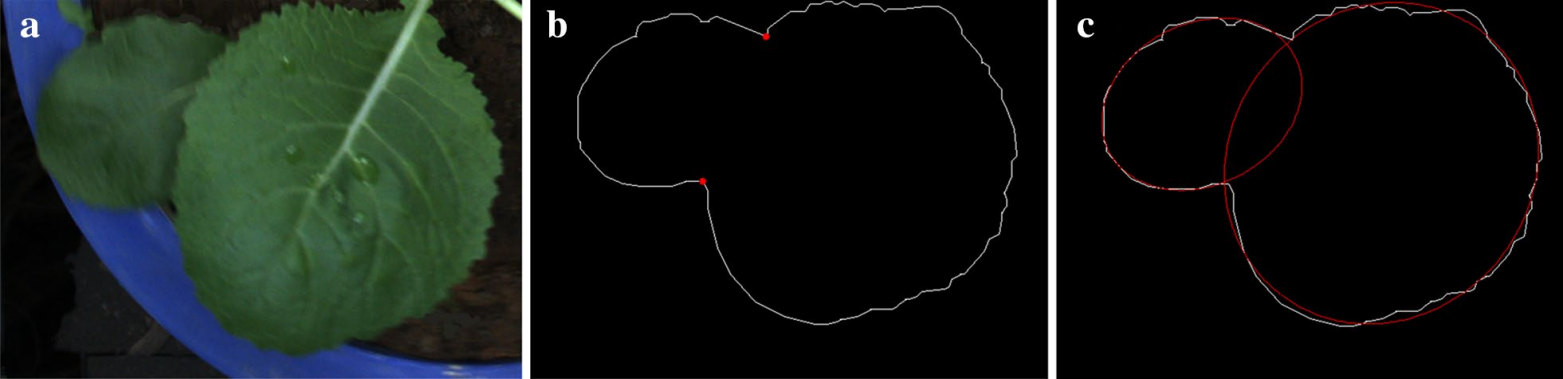
Recovering the overlapped leaf region for round-leaf. **a** The original overlapped round-leaf. **b** The concave points (*red*) and polygonal boundary (*white*) of overlapped round-leaf. **c** The ellipse fitting is chosen to recover the overlapped leaf region for round-leaf

The overlap of oilseed rape leaves is surely a difficult issue for binocular stereo-imaging. In this study, the oilseed rapes are at the seedling stage, which have less overlapping situation. Actually, for some situation (round-leaf), the overlap can be solved and recovered. The following part only considers the round-leaf (Fig. 9a). The detailed implementation steps are as follows: In the first step, we need to segment the overlapped leaf binary image. Secondly, the contour of overlapped leaf is extracted by using the front binary image. Then, the polygonal approximation [59] is used to represent the overlapped contour. This is an important step to trim away the small-scale rough fluctuations. Next, we need to detect the concave points [60] and segment the polygonal contour (Fig. 9b). Finally, the ellipse fitting [61] is chosen to recover the overlapped leaf region for round-leaf (Fig. 9c). The detailed algorithm description can refer to Additional files 6 and Additional File 7: Figure S5. The key for above algorithm is based on a priori knowledge: the oilseed rape leaf is approximate circle. Thus, for mosaic-leaf and semi-mosaic-leaf, the above method is useless.

## Conclusion

In this study, we establish a nondestructive and high-throughput stereo-imaging system for screening leaf canopy three-dimensional structure and individual leaf phenotypic traits. Compared with manual measurements, the squares of the correlation coefficients (*R* ^*2*^) for leaf area and plant height are 0.984 and 0.845, respectively. Moreover, 19 morphological traits were applied in morphology classification of three different rape leaf shapes. Three classifiers (SDA, SVM, and RF) were used and compared, and the better classification accuracy with SVM is 94.7% for 399 test samples. In conclusion, we developed a high-throughput stereo-imaging system to quantify leaf area, plant height, and leaf shape with more accuracy, which will benefit rape phenotyping, functional genomics, and breeding.

## Methods

### Plant materials and measurements

In total, 801 *Brassica napus* with three different shapes, including mosaic-leaf, semi-mosaic-leaf and round-leaf (Additional file 8: Figure S2), were analyzed in this study. Seeds were sown and germinated, and plants were grown up to the seedling stage. All plants were cultivated in plastic pots of 23.5 cm diameter with approximately 6 L of experimental soil. All pots were randomly distributed over a glasshouse compartment to control the growth conditions. Approximately 30 days after sowing, three experienced agronomists recorded the leaf shape using the visual method. The final statistical classification result would abide by the majority rule. All the experimental samples were measured with our stereo-imaging system to obtain image pairs. Among them, 66 rape plants were randomly selected to reconstruct the canopy three-dimensional structure, extract leaf area and calculate plant height. To estimate the accuracy of measurement, the plant leaf area was measured with the HLS [25] and plant height was measured manually by well-trained worker. In order to evaluate the extraction of individual leaf traits, a biological classification for three different leaf shapes was proposed. All samples were divided into two groups: one group consists of 402 samples with three labels (mosaic-leaf, semi-mosaic-leaf and round-leaf) as the training group. The other group consists of 399 samples without labels as the testing group. All the training group samples are selected randomly to balance the number of different shapes.

### Image analysis for canopy three-dimensional reconstruction

**Fig. 10:**
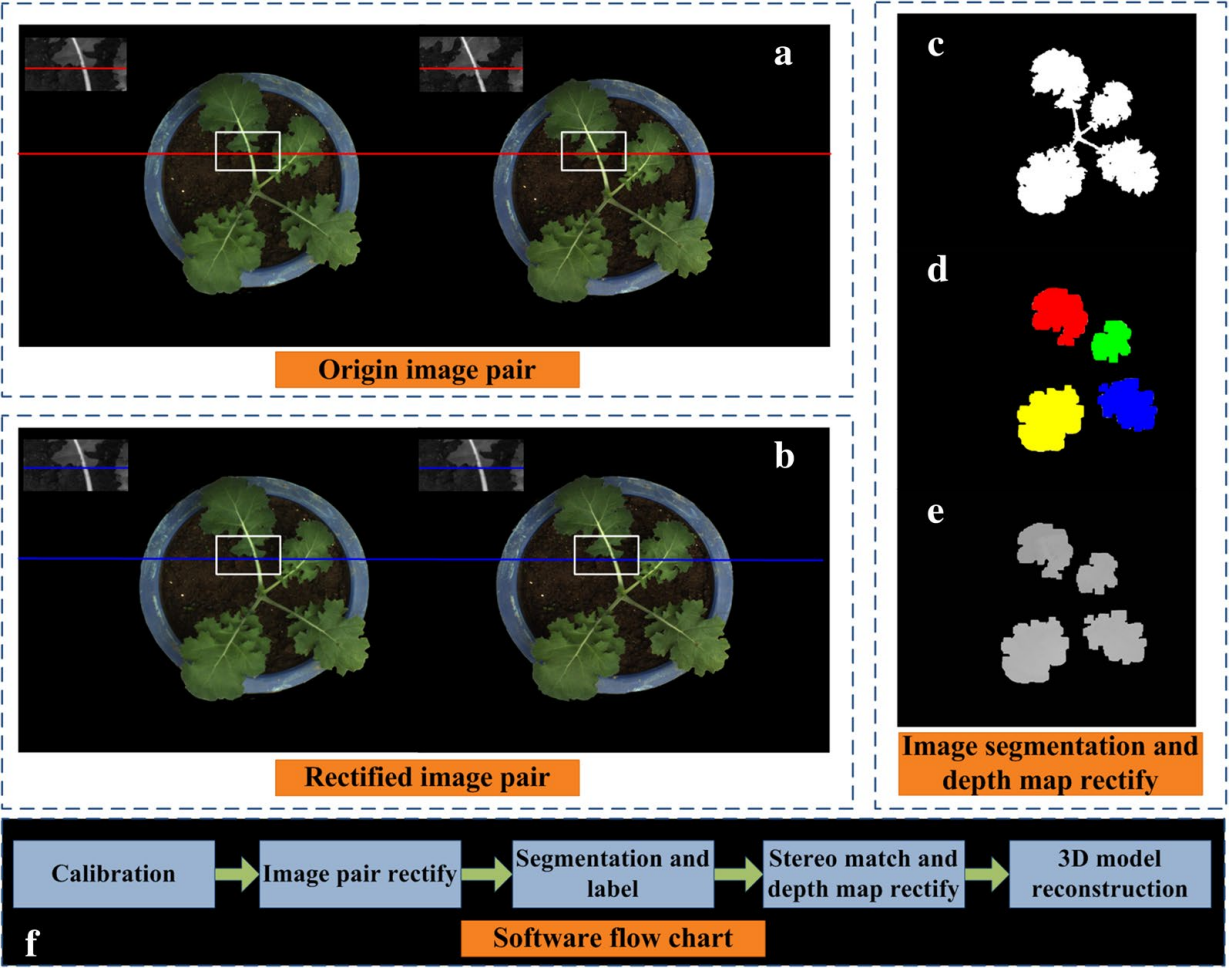
The primary image analysis procedures for seedling rape canopy three-dimensional reconstruction. **a** The same feature region in the left and right original image was not on the same horizontal baseline. **b** The same feature region in the left and right rectified image was at the same horizontal baseline. **c** The segmentation of rape leaf region. **d** The stems are removed, and the leaf region is marked. **e** A local rectification for discontinuous regions and false matching regions. **f** The main software flow chart

The main content of this part is to describe the specific image processing steps of canopy 3D reconstruction for seedling rape. The first step is camera stereo calibration. To achieve this step, a black and white calibration pattern [31] pasted on a plastic plate was used to obtain 20–25 image pairs. To ensure the accuracy of the calibration, the imaging angles should have obvious differences. For original image pairs (Fig. 10a), the corresponding feature in the left and right original image is not on the same horizontal baseline. Here, Bouguet algorithm [62] was used to rectify two original images. The final rectified image pairs were shown in Fig. 10b. Considering the influence of environmental light, an automatically segmenting method [63], adopts normalized RGB component to get binary image of blade region (Fig. 10c). Considering the slender characteristics of the stem, the morphological opening operation is used to remove the stem, and connected component mark technology is used to distinguish different leaves (Fig. 10d). The next work is to match the corresponding feature points in the left and right rectified images. Here, the library for efficient large-scale stereo matching [64] is used to compute the left and right disparity map. In the actual situation, two thorny situations might happen [65, 66]. The first one is mismatch, which means that there are no matching pixels or wrong matching pixels. The second thorny situation is occlusion, which means that some pixels appears only in an image, and can’t see in another image (Additional file 9: Figure S6). If we don’t take some special measures to focus on region where the mismatched and occluded situations are serious, there will have some wrong in the process of 3D point clouds extraction. So, it is important to rectify the disparity map. The specific process is described in Additional file 10 and the rectified left disparity image is shown in Fig. 10e. According to the principle of triangular range (Additional file 11: Figure S1), we can extract the three-dimensional point cloud data of the canopy leaves. After removing the isolate points, triangle patches are used as the surface of canopy leaves by using Delaunay triangulation algorithm [50]. The final result of canopy reconstruction is shown in Fig. 2b–d. Detailed processing for canopy 3D reconstruction and triangle patches generation has been described in Additional file 10 and Fig. 3. The code for Delaunay algorithm is shown in Additional file 12.

### Image analysis for individual leaf

**Fig. 11:**
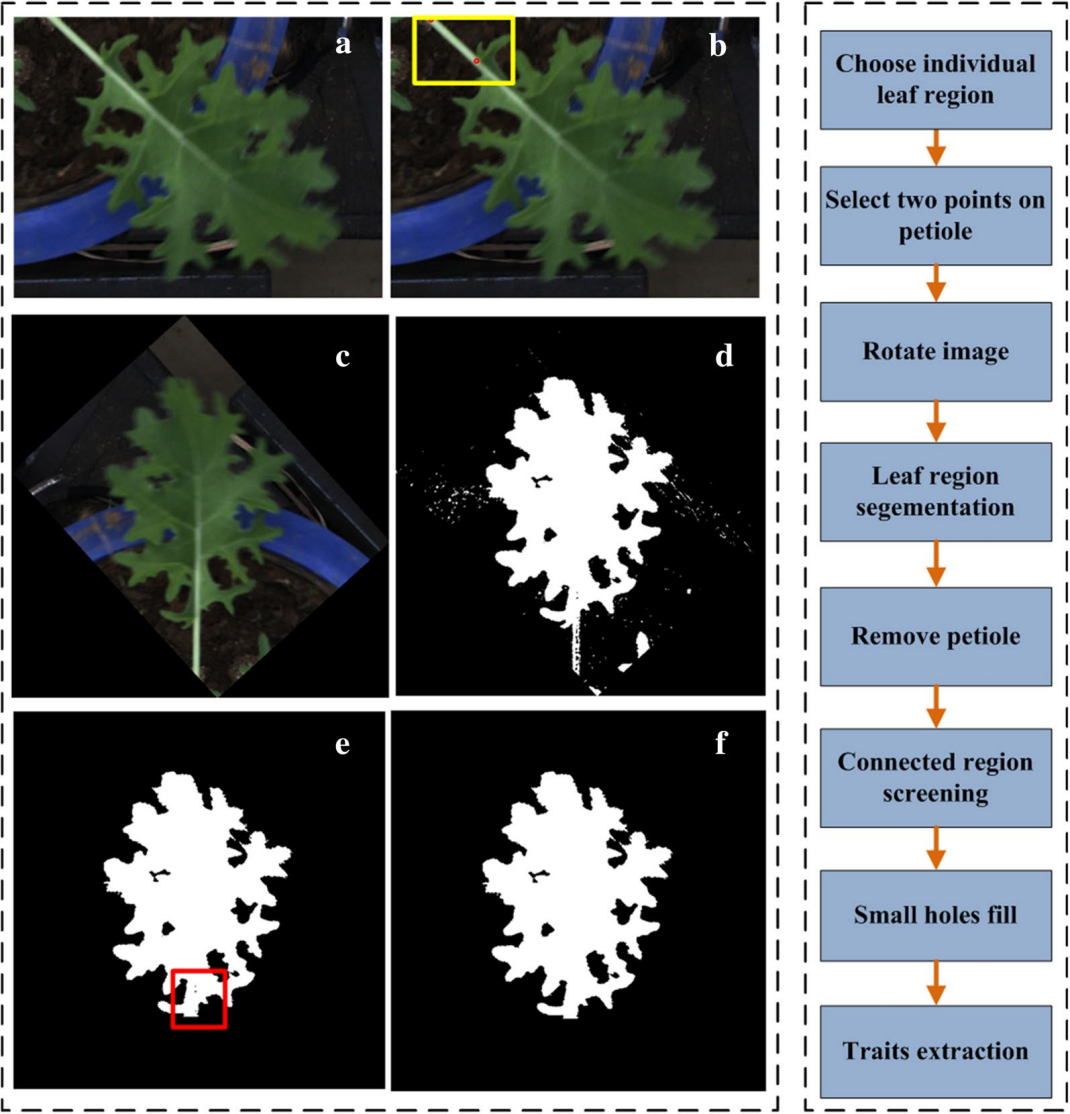
The primary procedure for individual leaf traits extract. **a** The original rape leaf image. **b** The selected single rape leaf region. **c** Select two points on the image. The first point is the intersective region of stem and leaf and the second point is used to decide the direction of rotation. **d** The rotated oilseed rape leaf image. **e** Using normalized EG and ER to segment the leaf region. **f** Remove the petiole region and connected region screening

The main content of this part is to describe the specific image processing steps for individual leaf extraction. Firstly, the user needs to click the left mouse button and drag it to choose a rectangular box. In this rectangular box, the individual leaf must be typical and representative (Fig. 11a). All selected rectangular images are saved in PNG format for subsequent analysis. Usually, the selected individual leaf has a long petiole part, the existence of which will seriously impact the analysis of blade traits. So, the next step is to remove petiole. The difference between blade and petiole in color and texture is tiny. But from the view of shape, petiole region is more slender than blade. With that mechanism we can remove petiole. The detailed operating processing includes the following steps: (1) Marking two points on the petiole (Fig. 11b) and rotating the rectangular image so that the direction of the petiole is downward (Fig. 11c). (2) Segmenting rotated rectangular image to obtain binary leaf image (Fig. 11d). Here, the excess green vegetation (*ExG*) [67] and excess red vegetation (*ExR*) indices [68] were used to extract binary leaf image. (3) From the bottom to top search binary image to remove the pixel width less than a specified threshold area (Fig. 11e). Here, the area threshold is set to 25, which is an appropriate value determined by lots of preliminary experiments. After removing the petiole, the next step is to remove connected components that were erroneously selected. The situation that other partial leaf region might be chosen in the rectangular region was always happened. Usually, the target leaf region had the largest area. So, only thing we need to do is to keep the largest connected component as the target individual leaf. In addition, because of the binarization segmentation error, small holes might appear on the target blade region (the red square in Fig. 11e). Usually, these holes have a small number of pixels compared with other real holes. An area threshold is used to fill the small area holes. The final individual leaf is shown in Fig. 11f. The detailed processing flow and computational formulas are in Additional file 13.

### Three different classification methods

#### Stepwise discriminant analysis statistical method

For stepwise discriminant analysis (SDA), the specific operating approaches are as follows: All traits are selected as the input variables of the algorithm. Then, the SDA algorithm will select a variable that has the most significant discriminant ability. Next, the selecting for second variable based on the first variable, which indicates that combining the first and second variables will have the most significant discriminant ability. By that analogy, the third variable will be selected. Because of the mutual relationship between different variables, the previous variable may lose significant discriminant ability after inputting the new variable. Then, we will inspect the discriminant ability of all previous selected variables to find the disabled variables, remove them, and go on to find new variables until no significant variables can be removed. In this study, stepwise discriminant analysis training was achieved using (SPSS v.22 software), which is a proven technique for meaningfully classifying different shapes [69]. The detailed description is shown in Additional file 14.

#### Support vector machine statistical method

The support vector machine (SVM) is a common supervised learning algorithm that has been shown to provide state-of-the-art performance in many classification problems. The main thinking of the SVM is to establish a classification hyperplane as the decision curved surface, which maximizes the gap of positive samples and negative samples. The LIBSVM-matlab toolkit [70] was used here to conduct SVM model. All the 801 samples with three different leaf shapes were randomly divided into two groups (402 samples for training and 399 samples for testing). Firstly, we should limit all data into a certain range. Here, the interval from 0 to 1. The purpose for data normalization [71] is to ensure the convergence of the SVM algorithm. At the same time, it will improve the accuracy of classification. Next, we can train the model. Here, the kernel function is generated to use polynomials and the kernel parameter is set to 1.5. Also, the penalty parameter is set to 2. The genetic algorithm (GA) was used to choose the best value of kernel parameter and penalty parameter. The detailed algorithm process refers to Additional file 14 and the code is shown in Additional file 15.

#### Random forest statistical method

The random forest (RF) classifier is a combination of multiple decision trees. In this study, the open source randomforest-matlab toolkit [72] was adopted to build random forest classifier. Abhishek Jaiantilal, of the University of Colorado, Boulder, is the primary developer. Here, the number of decision trees in my random forest is 1000 and the other parameters adopt the default value. 801 samples were randomly selected and divided into two groups: 402 samples comprising the training group and 399 samples for testing group. When the test samples enter into the random forest, every decision tree will independently classify the category it belongs to. The final statistical classification result will abide by the majority rule. The detailed algorithm process refers to Additional file 14 and the code is shown in Additional file 16.

## Additional files

**Additional file 1: Figure S3.** The software interface for seedling rape canopy three-dimensional reconstruction.

**Additional file 2.** Original data of leaf area and canopy height.

**Additional file 3.** Original data of individual leaf traits.

**Additional file 4: Figure S4.** The software interface for extracting individual leaf traits.

**Additional file 5.** Supplementary document for manual interaction.

**Additional file 6.** Supplementary document for overlap recovery algorithm.

**Additional file 7: Figure S5.** Overlap recovery algorithm flow.

**Additional file 8: Figure S2.** Three different shapes of rape leaf in seedling stage.

**Additional file 9: Figure S6.** The occluded situation in binocular stereo-imaging system.

**Additional file 10.** Supplementary document for three-dimensional reconstruction.

**Additional file 11: Figure S1.** The triangle range finding for optical path.

**Additional file 12.** Supplementary document for Delaunay code.

**Additional file 13.** Supplementary document for individual leaf traits.

**Additional file 14.** Supplementary document for three classification methods.

**Additional file 15.** Supplementary document for SVM code.

**Additional file 16.** Supplementary document for RF code.

## Abbreviations

AA: the aspect ratio of leaf minimum circumscribed box
R: the ratio of leaf area to minimum circumscribed box area
AC: the ratio of leaf area to leaf convex hull area
PC: the ratio of leaf circumference to leaf convex hull perimeter
S: the ratio of leaf area to the square of leaf convex hull perimeter
E: the ratio of long axis of ellipse to short axis of ellipse
FF: the ratio of leaf area to the square of leaf perimeter
PAR: the ratio of leaf circumference to leaf area
SFD: reflect the effectiveness of the occupies space without image cropping
IFD: reflect the effectiveness of the occupies space with image cropping
C: the ratio of inscribed circle radius to circumscribed circle radius
NIC: the number of inner cavities
APIC: the average perimeter of inner cavities
AAIC: the average area of inner cavities
TNI: total number of indents
ENI: effective number of indents
ADI: the average depth of indents
ADEI: the average depth of effective indents
AVE: the average calculated value of effectiveness
SDA: stepwise discriminant analysis
SVM: support vector machine
RF: random forest
GA: genetic algorithm
