# Supplementary figures and images for: A high-throughput stereo-imaging system for quantifying rape leaf traits during the seedling stage
Source: Plant Methods. 2017 Jan 31;13:7. doi: 10.1186/s13007-017-0157-7 (PMC5282657; doi:10.1186/s13007-017-0157-7)

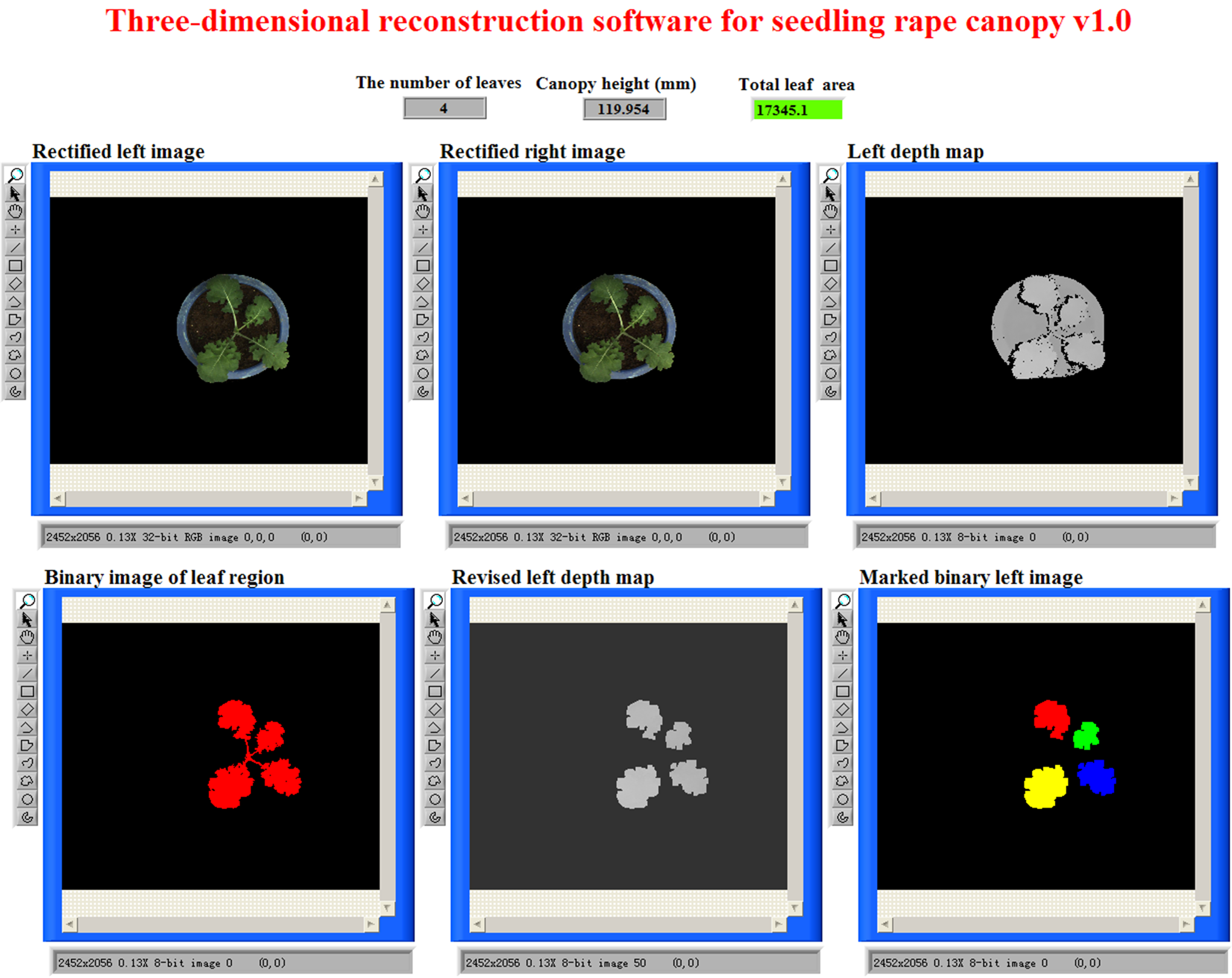

Supplement: Supplementary file 1 — Additional file 1: Figure S3. The software interface for seedling rape canopy three-dimensional reconstruction. [file 13007_2017_157_MOESM1_ESM.tif]

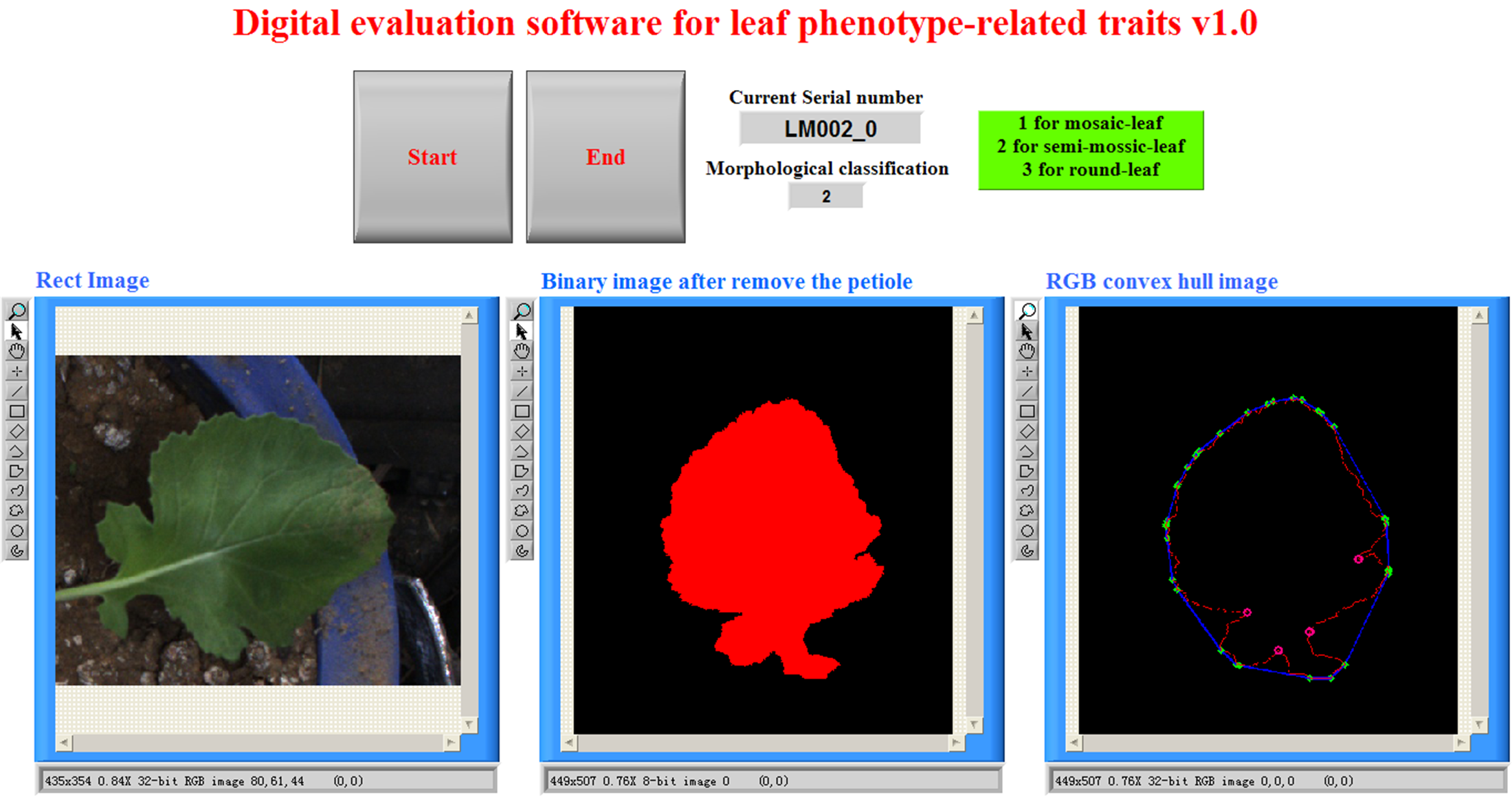

Supplement: Supplementary file 4 — Additional file 4: Figure S4. The software interface for extracting individual leaf traits. [file 13007_2017_157_MOESM4_ESM.tif]

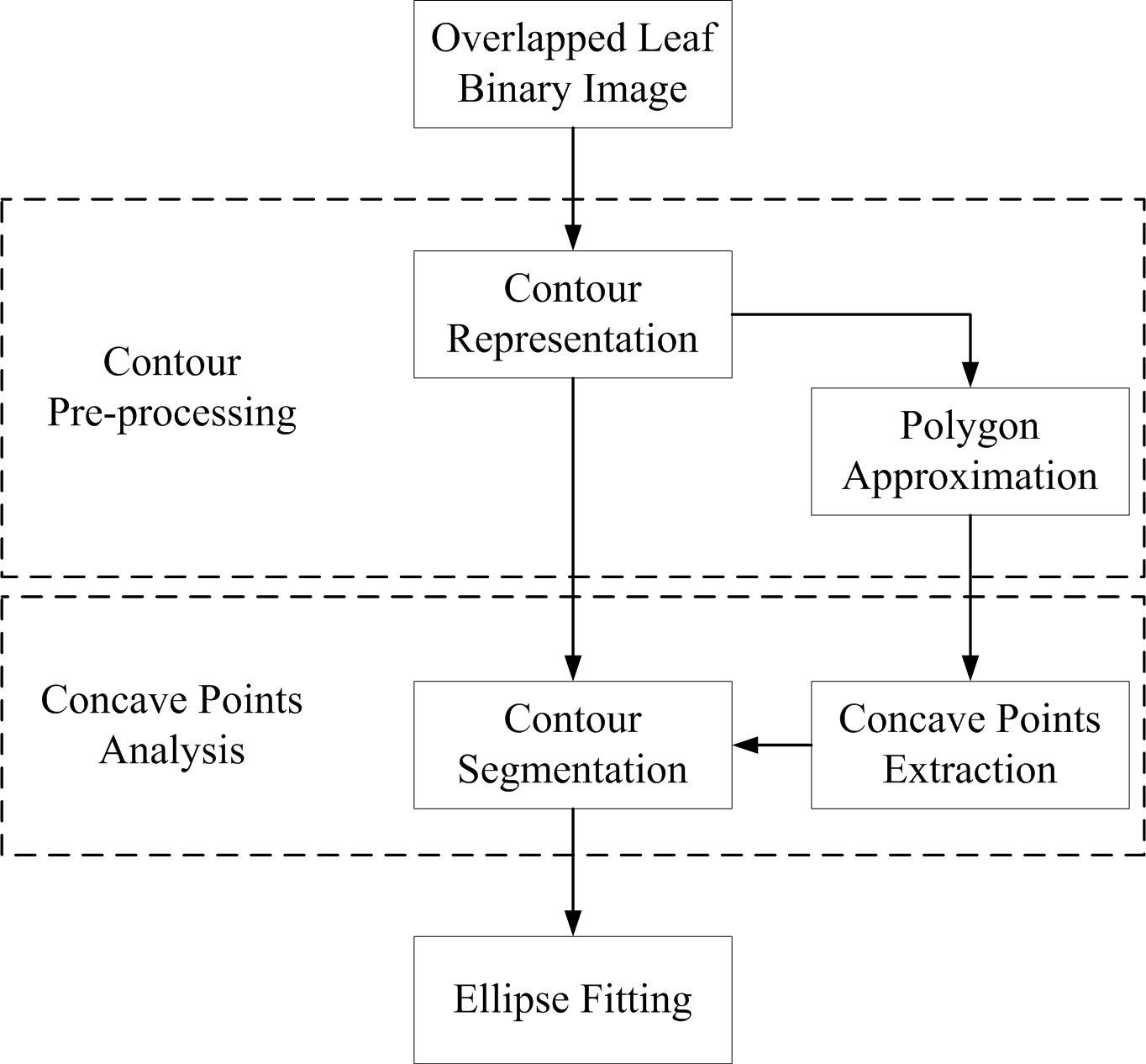

Supplement: Supplementary file 7 — Additional file 7: Figure S5. Overlap recovery algorithm flow. [file 13007_2017_157_MOESM7_ESM.tif]

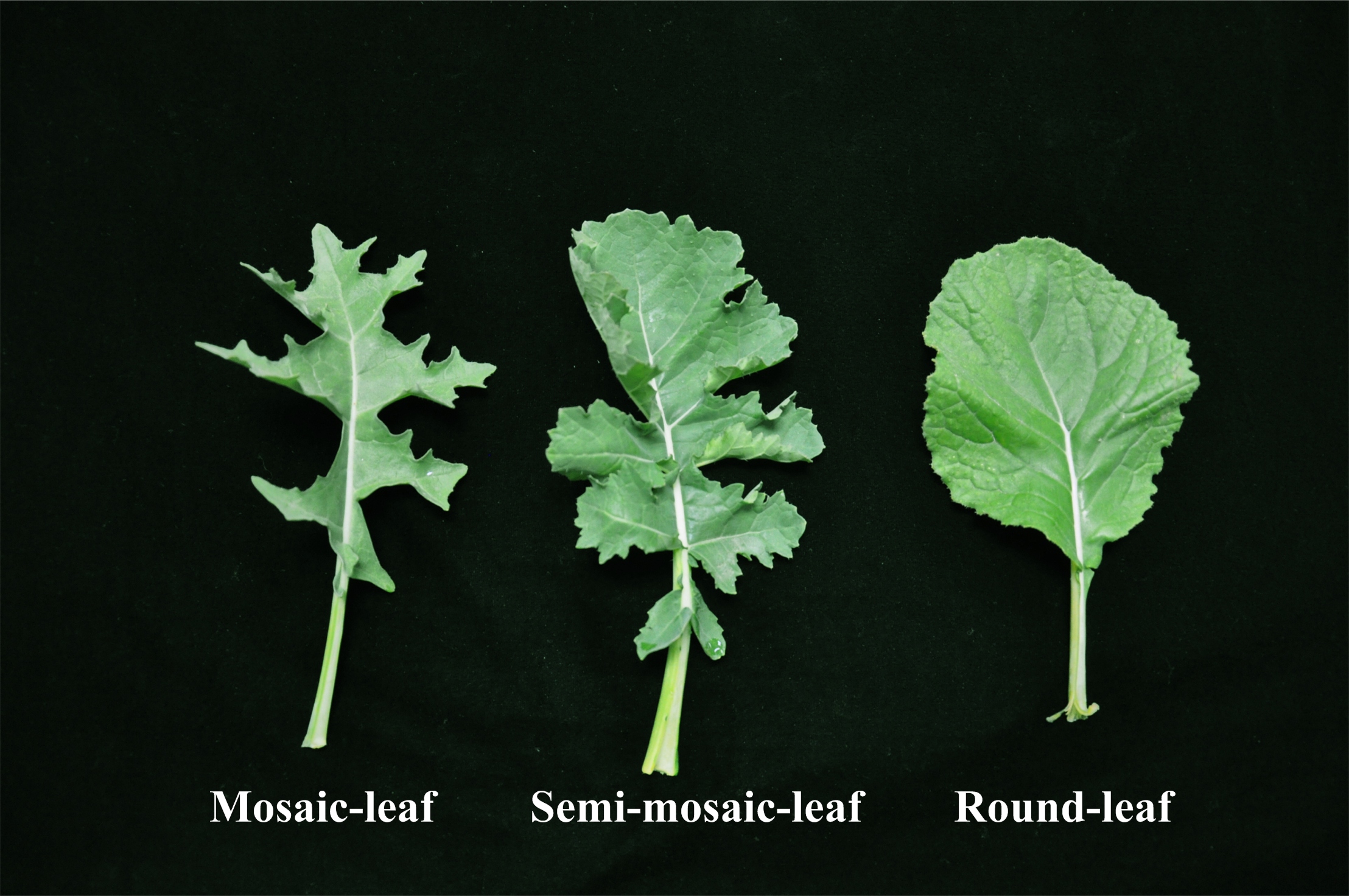

Supplement: Supplementary file 8 — Additional file 8: Figure S2. Three different shapes of rape leaf in seedling stage. [file 13007_2017_157_MOESM8_ESM.tif]

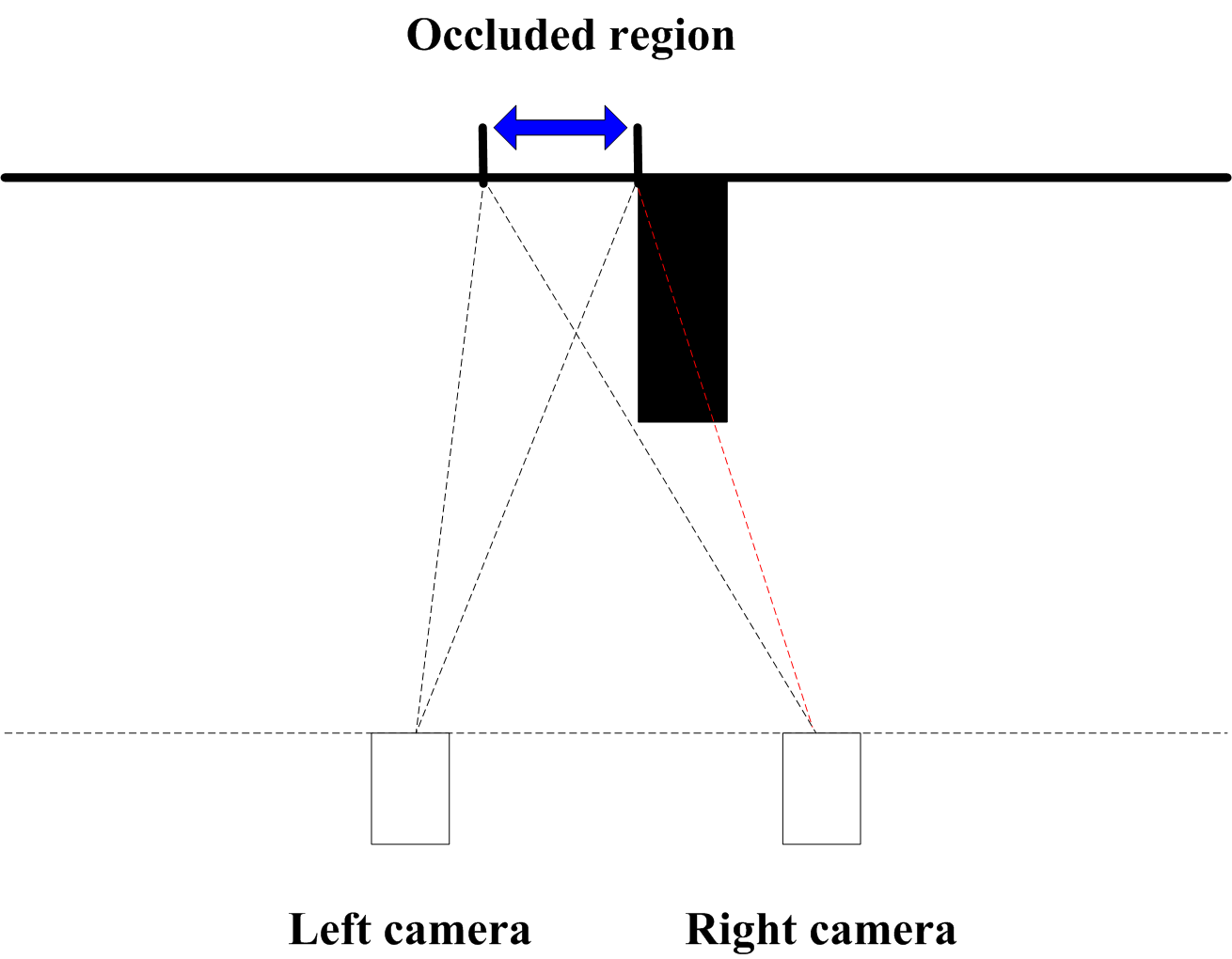

Supplement: Supplementary file 9 — Additional file 9: Figure S6. The occluded situation in binocular stereo-imaging system. [file 13007_2017_157_MOESM9_ESM.tif]

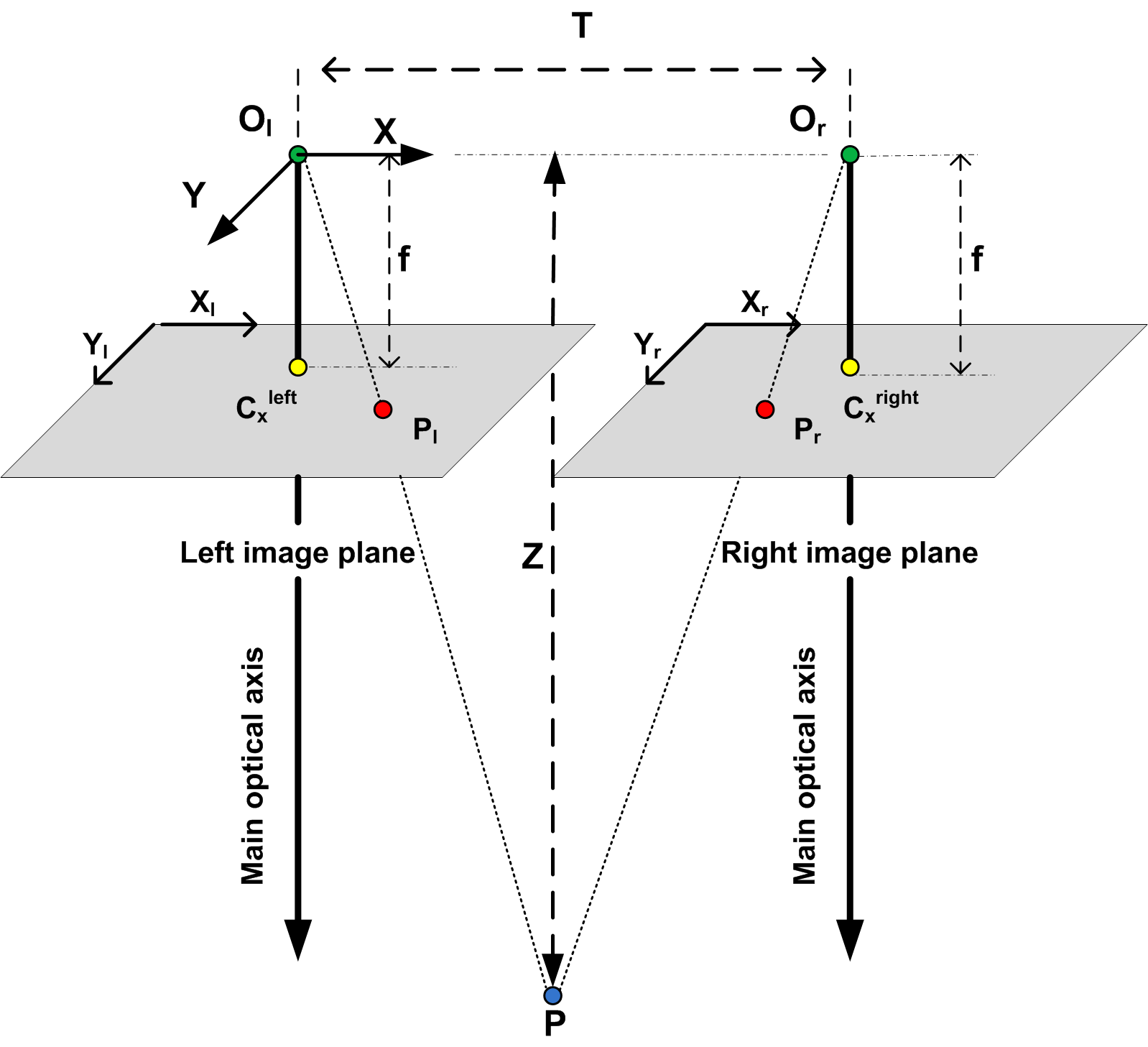

Supplement: Supplementary file 11 — Additional file 11: Figure S1. The triangle range finding for optical path. [file 13007_2017_157_MOESM11_ESM.tif]
