# Supplementary material for: A high-throughput stereo-imaging system for quantifying rape leaf traits during the seedling stage
Source: Plant Methods. 2017 Jan 31;13:7. doi: 10.1186/s13007-017-0157-7 (PMC5282657; doi:10.1186/s13007-017-0157-7)
Supplement: Supplementary file 6 — Additional file 6. Supplementary document for overlap recovery algorithm. [file 13007_2017_157_MOESM6_ESM.doc]

**Overlap recovering algorithm**

The main content of this part is to describe the theorys and specific implementation for overlap recovering algorithm. The overlap of oilseed rape leaves is surely a difficult issue for binocular stereo imaging. Actually, for some situation (round-leaf), the overlap can be recovered. The following part only considers the round-leaf, we can recover the overlapped region. The detailed implementation steps are as follows:

In the first step, we need to extract the overlapped leaf binary image. An automatically segmenting method, which adopts the normalized RGB component to segement foreground green pixels. Detailed computational expressions are as follows 1-4:

$R=\frac{r}{(r+g+b)}\text{ }G=\frac{g}{(r+g+b)}\text{ }B=\frac{b}{(r+g+b)}$ (1)

$ExG=2G-R-B\text{ > Threshold\_G ? 1 ,0}$ (2)

$ExR=1.4R-G>\text{ Threshold\_R ? 1 , 0}$ (3)

$bw=ExG-ExR$ (4)

Where *r*, *g* and *b* indicate the pixel values of the red, green and blue channels, respectively. Here, the segmentation algorithm adopts the same method in Additional File 9. Then, the contour of overlapped leaf can be extracted through various algorithms, such as thresholding algorithm, watershed transform, edge detection and so on. In this study, the edge detection function “findContours” in OpenCV tookit was used to extract external contour of overlapped region. The detailed algorithm process for overlapping recovery is shown below (Figure 1).


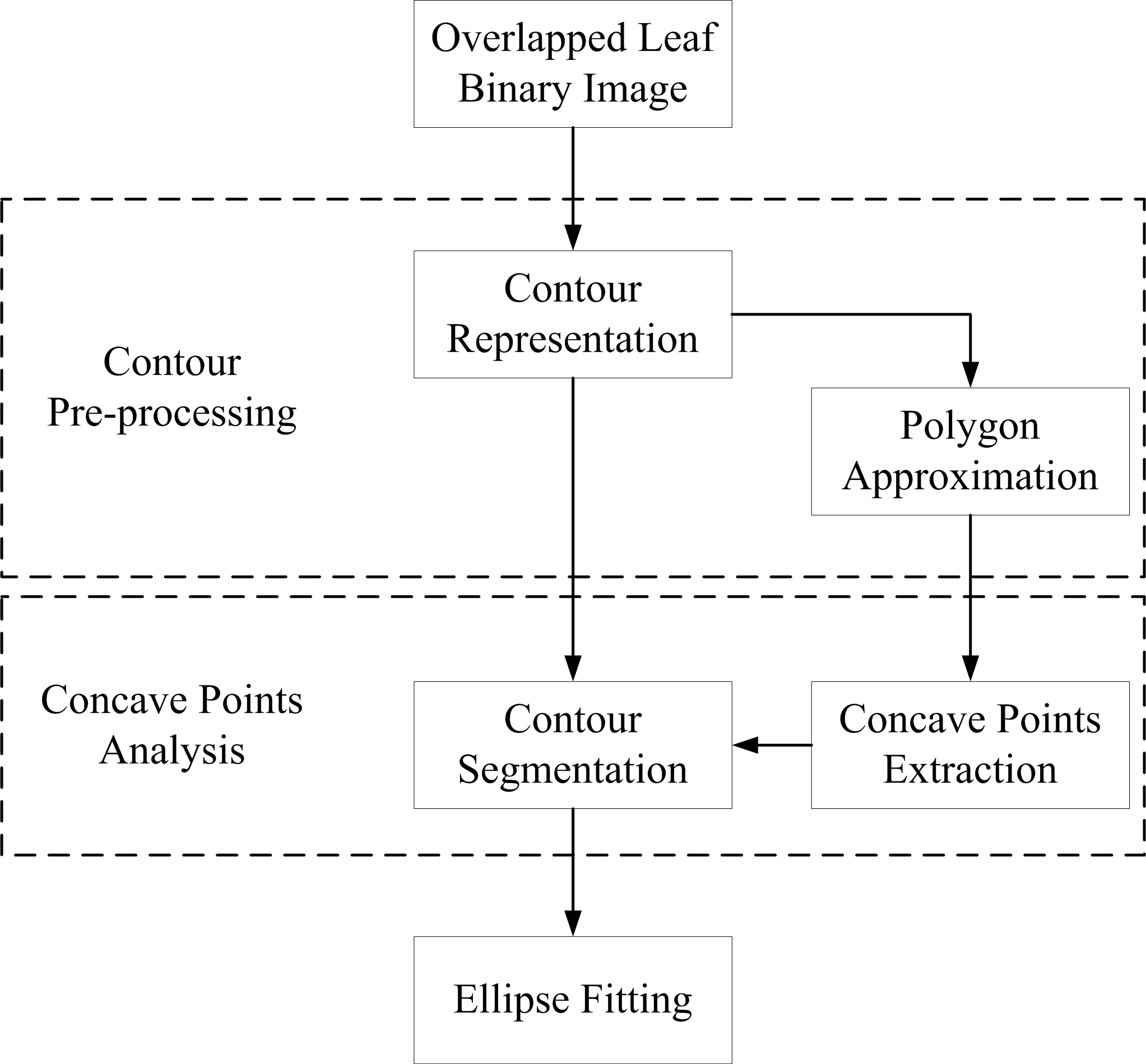


Figure 1. Overlap recovery algorithm flow.

The contour of overlapped leaf is then represented by a sequence of points. The original contour may be rough and has many small-scale fluctuations which affect the calculation of the concavity and may cause many false concave points. Thus, the polygonal approximation algorithm [1] is used to represent the overlapped contour. This is an important step to trim away the small-scale rough fluctuations. A brief description of polygon approximation algorithm is the following: Suppose there are *N* points in the original contour represented. The procedure of this algorithm starts from two points: *p_i_* and the next nStep point *p_j_* .(nStep represents the number of points between *p_i_* and *p_j_*) The next step is calculating the distances from all the points of the contour between *p_i_* and *p_j_* to the line *p_i_p_j_* , and comparing these distances with a pre-set threshold *d_Th_*. If there is a point *p_t_* whose distance *dt ≥ d_Th_*, then *p_t_* is a point of the contour to be kept after polygon approximation, and *p_i_* will move to *p_t_* to repeat the previous procedures. Otherwise *p_j_* moves to the next point. Obviously, small value of *d_Th_* gives a precise polygon approximation [1].

Next, we need to detect the concave points (Figure 2b). In this study, the CPDA [2] technique is then used to estimate the curvature on the smoothed contour. In order to make the strong and weak corners more distinguishable, the CPDA detector ﬁrst uses three different chord-lengths to estimate three normalized discrete curvature values on each point of the smoothed curve. It then multiplies the normalized curvature values to obtain the curvature product (a single estimated curvature) at each point. The maxima of the absolute curvature products along the smoothed curve are then marked as candidate corners. Finally, it follows a two-step reﬁnement process that uses a curvature-threshold and an angle-threshold to remove any weak and false corners respectively.

According to the position of concave points, we can segment the smoothed contour into different subsections. Finally, the ellipse fitting [3] is chosen to recover the overlapped leaf region for round-leaf (Figure 2c). Usually, the methods of ellipse fitting can be divided into two classes: clustering and least square fitting. The methods of least square fitting are computationally cheap and perform well in most cases. One widely used least square fitting method, namely the direct least square method. Here, we use this fitting method to recover overlapped leaf.


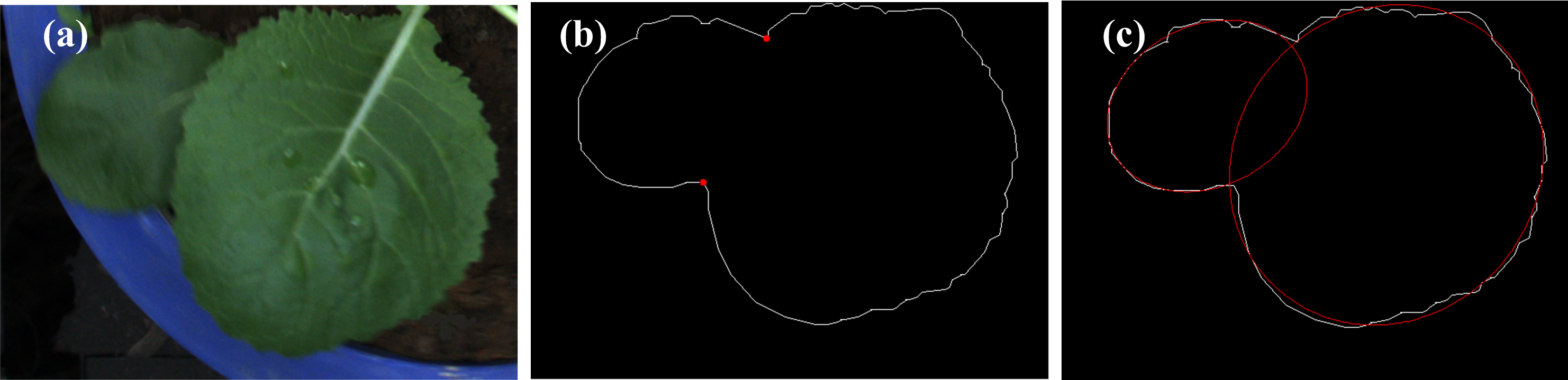


Figure 2. Recovering the overlapped leaf region for round-leaf

The key for above algorithm is based on a priori knowledge: the oilseed rape leaf is approximate circle. Thus, for mosic-leaf and semi-mosic-leaf, the above method is useless.

**References**

[1] Bai X, Sun C, Zhou F. **Splitting touching cells based on concave points and ellipse fitting ☆**. Pattern Recognition, 2009, 42(11):2434-2446.

[2] Awrangjeb M, Lu G, Fraser C S, et al. **A Fast Corner Detector Based on the Chord-to-Point Distance Accumulation Technique. Digital Image Computing: Techniques and Applications.** IEEE Computer Society, 2009:519-525.

[3] White N D G, Jayas D S, Gong Z. **Separation of Touching Grain Kernels in an Image by Ellipse Fitting Algorithm.** Biosystems Engineering, 2005, 92(2):135-142.
