# Supplementary material for: A high-throughput stereo-imaging system for quantifying rape leaf traits during the seedling stage
Source: Plant Methods. 2017 Jan 31;13:7. doi: 10.1186/s13007-017-0157-7 (PMC5282657; doi:10.1186/s13007-017-0157-7)
Supplement: Supplementary file 10 — Additional file 10. Supplementary document for three-dimensional reconstruction. [file 13007_2017_157_MOESM10_ESM.doc]

**Three-dimensional reconstruction for seedling rape canopy**

The main content of this part is to describe the specific steps of three-dimensional reconstruction for seedling rape canopy and major image processing technologies. All of these image processing technologies are completed by the computer automatically. The detailed description as follows:

**Camera calibration**

In general, the first step before acquiring images is camera calibration. The purpose of calibration is to extract the intrinsic parameters, distortion parameters and extrinsic parameters.

In an ideal pinhole camera model，the process of each point in real world map to projection plane is called projection transformation. In this process, each three-dimensional point will follow the Equations 1-2 [1].

$x_{screen}=f_{x}\left(\frac{X}{Z}\right)+c_{x}$ (1)

$y_{screen}=f_{y}\left(\frac{Y}{Z}\right)+c_{y}$ (2)

Where, *(X,Y,Z)* are the coordinates of a three-dimensional point in the world coordinate system. *(x_screen_,y_screen_)* are the coordinates of the projection point in the image plane. *(c_x_,c_y_)* are the coordinates of the principal point of the camera in the image plane. *(f_x_,f_y_)* are the focal lengths expressed in pixel units.

Here, we introduce two different focal lengths. The reason is that each pixel in imaging plane stands a good chance of not a square. So, the focal length *f_x_* actually means the product of lentiform physical lens focus length F and the imager horizontal unit size *s_x_*. The same reason also applies to *f_y_* and *s_y_*. Also, due to the camera installation accuracy problem, the principal point will not keep consistent with the center of the imaging plane. Hence, the four related paramter *(f_x_,f_y_,c_x_,c_y_)* which were called intrinsic camera parameters need to be calculated by camera calibration.

In real life, the amount of light can hardly meet the actual demand by using the ideal pinhole camera model, which will make the imaging speed very slow. In order to solve this problem, optical lens with large area were used to gather more light to meet the imaging speed demand. Because of this, the distortions will be inevitable introduced into imaging process. Usually, the len distortions consist of two marjor parts: the radial distortion and slightly tangential distortion. Radial distortion always comes from the shape of the lens, and the tangential distortion from the whole assembly process of the camera. The expressions are shown below:

$x_{corrected}=x\left(1+k_{1}r^{2}+k_{2}r^{4}+k_{3}r^{6}\right)+2p_{1}y+p_{2}\left(r^{2}+2x^{2}\right)$ (3)

$y_{corrected}=y\left(1+k_{1}r^{2}+k_{2}r^{4}+k_{3}r^{6}\right)+2p_{2}x+p_{1}\left(r^{2}+2y^{2}\right)$ (4)

Where, *(x,y)* are the original coordinates of the distortion point in imaging plane. *(x_corrected_,y_corrected_)* are the new coordinates of the distortion point after rectifing. *(k_1_,k_2_,k_3_)* is the coefficient of radial distortion. *(p_1_,p_2_)* is the coefficient of tangential distortion.

Moreover, the extrinsic camrea parameters are rotation and translation angles transformed from the camera coordinate system to the world coordinate system [2]. So, the purpose of calibration is to extract these parameters accurately. Here, a black and white calibration pattern [3] pasted on a plastic plate was used. By using the two top-view cameras, nearly 20-25 image pairs with different camera angles would be obtained. The calibration processing needed to be performed only once prior to image analyses. To keep the accuracy of the calibration, the camera angles should have obviously difference and the angle range should cover almost whole directions. Then, the corner points were extracted from calibration pattern as the detectable features to calculate intrinsic matrix of the camera and the distortion coefficients of the lens. Here, the camera calibration toolbox [4] for matlab was choosen as the main calibration tool to extract all related calibration parameters.

**The rectification of image pair**

In the practical research, the two cameras were aligned in a fixed geometry. While, no matter how we parallel to put the two cameras in real world, there are still have some difference in angle and distance. The corresponding feature in left and right original image was not on the same horizontal baseline. So, the purpose of this step is to eliminate the distortion and reproject two images to make the two imaging planes at the same level. Here, Bouguet algorithm [4] was used to ensure the maximizing views overlap and minimizing distortion. In the actual operation, the OpenCV (Open Source Computer Vision Library) tookit was used to deal with the rectify problem. The OpenCV tookit was an open source image processing algorithm library and the core algorithms in it were developed in C and C++ language. We can call the image processing functions in it by adding them to C++ program. The Bouguet algorithm which was encapsulated in it was selected to complete the distortion correction and line alignment. Here, the intrinsic parameters and distortion parameters, extracting from the step of camera callbration, were applied as the input parameters. The final rectified image pairs were shown in Figure 10b, and the white rectangle regions were demonstrated in the upper part of image.

**Leaf segmentation and label**

Here, an automatically segmenting method was used to extract canopy leaf region. Due to the influence of environmental light shooting, the surface of rape leaf was uneven. Addition, the petiole region was more whitter than blade region, which will make foreground pixels extraction incompletely. Considering the previous reasons, the excess green vegetation index (*ExG*) [5] and excess red vegetation index (*ExR*) [6] was used in the image segmentation. In stead of traditional RGB (red, green and blue) color component extraction, this new algorithm [7] adopts the normalized RGB component to segement foreground pixels. Computational expressions are as follows 5-8:

$R=\frac{r}{(r+g+b)}\text{ }G=\frac{g}{(r+g+b)}\text{ }B=\frac{b}{(r+g+b)}$ (5)

$ExG=2G-R-B\text{ > Threshold\_G ? 1 ,0}$ (6)

$ExR=1.4R-G>\text{ Threshold\_R ? 1 , 0}$ (7)

$bw=ExG-ExR$ (8)

Where *r*, *g* and *b* indicate the pixel values of the red, green and blue channels, respectively.

The leaf extraction process began with an excess-green minus excess red (ExG−ExR) to remove residue background. Usually, ExG can separate most of the plants. However, the segmentation process may fail when the existence of green–red material, such as plant stem branches and petioles. Moreover, the extraction of ExR component improved the process by eliminating red–green regions. Usually, the red–green regions represent organic residue, which might interpreted as plant material [8]. After achieving the above steps, the target regions were segemented into binary image (Figure 10c). In this way, we can put this binary image as a mask to calculate the three-dimensional structure of rape plant’s canopy. In order to more accurately assess the leaf area, stem regions need to be removed from the binary image. Considering the slender characteristics of the stem, a morphological opening operation was used. Finilly, the connected component mark technology was used to distinguish different leaves into different colors (Figure 10d).

**Stereo match and disparity rectify**

The next work is to match the corresponding feature points in the left and right rectified images. After the previous steps, the corresponding feature points in the left and right were arranged in the same horizontal baseline. So, searching through the horizontal baseline is the only way we should do to find corresponding feature points. Here, we define the left depth and right depth as the following expressions 9-10:

$d_{left}=x_{left}-x_{right}$ (9)

$d_{right}=x_{right}-x_{left}$ (10)

Where, *x_left_* is the horizontal coordinates of the projection point in the left image plane. *x_right_* is the horizontal coordinates of the projection point in the right image plane

Here, the libelas (library for efficient large-scale stereo matching) [9] was used. The libelas is a cross-platfrom C++ library with Matlab wrappers for computing left and right disparity from rectified graylevel stereo pairs. This algorithm is robust against moderate changes in illumination and well suited for robotics applications with high resolution images. Computing the left and right disparity map of a one Megapixel image requires less than one second on a single i7 CPU core.

While, there are two thorny situations might happen [10-11] in the process of stereo matching. The first one is mismatch, which means that there are no matching pixels or wrong matching pixels. The reasons for this situation are many, including low texture, repetitive texture and photometric variation, etc. The second thorny situation is occlusion, which means that some pixels appears only in an image, and can't see in another image. So, if we don't take some special measures to focus on region where the mismatched and occluded situations are serious, there will have some wrong in the process of 3D point clouds extraction. So, it is important to rectify the left and right depth.

The specific rectified process is as follows: Firstly, find all the coordinates of points where the depth value is zero or the depth value bigger than a certain threshold. Secondly, take each point in turn, starting from this point, turn left and right to find the first point whose vlaue is not zero, then, take the larger of the two values as the final value. After the above steps, we can get rectified depth image pairs easily. The rectified left depth image is shown in Figure 10e. This method performs well to the occluded and mismatched regions and achieves more precise disparity maps, which reduce the emergence of outlier in the generation of 3D point clouds.

**3D model reconstruction**

According to the principle of triangular range (Addition file 1: Figure S1), we can obtain the three-dimensional point cloud of canopy leaves. A filter was firstly used to remove the isolate points. Then, the triangle patchs were choosen as the surface of the leaf target. Here, the Delaunay algorithm was used in the process of three-dimensional mesh generation. Delaunay is a usual algorithm to create Delaunay triangulations of a set of points in 2D/3D space. A Delaunay triangulation ensures that the circumcircle associated with each triangle contains no other point in its interior. In this study, the matlab functions “trimesh” and “delaunay” based on global method (Lifting Method) [12] are used to achieve Delaunay algorithm. Lifting Method is a widely used algorithm to construct Delaunay triangulations by using QHULL [13] to construct the convex hull. The detailed description for Lifting Method is shown below: 1. lift points in *R^d^* to the graph of the paraboloid in *R^d+1^* by summing the squares of their coordinates; 2. construct convex hull of lifted points in *R^d+1^*; 3. remove the vertical dimension from the lower envelope of the convex hull; 4. project the lower convex hull back to *R^d^*. So, QHULL is a good method to map the points in low dimension space to that of high dimension space and compute the convex hull in high dimension space. Then back again. In this way, you can calculate which points constitute a triangle. The "delaunay" function can achieve above function and produces an isolated triangulation, which is useful for applications like plotting surfaces via the "trimesh" function. The detailed Delaunay code can refer to Additional File 11 and the detailed process for leaf triangular patches generation is shown in manuscript Figure 3. If we want to calculate the area of leaf region, the rough surface obviously brings great area error. So, the smoothing mechanism (Figure 3b-c) which is based on triangle patchs was used to solve the problem. This smoothing mechanism can well guarantee the leaf size of the outer boundary, and also can change as the direction of the leaf. The final reconstruction leaf region is shown in manuscript Figure 2.

**References**

[1] Matsuura, Y., He, D. X. **A Binocular Stereovision System for Transplant Growth Variables Analysis**. Applied Engineering in Agriculture, 2003, 19 (5): págs. 611-618.

[2] Hartley, R., Zisserman, A. **Multiple view geometry in computer vision**. Cambridge University Press, 2006, 30 (9-10): 1865 - 1872.

[3] Baumberg, A., Lyons, A., Taylor, R. **3D S.O.M.—A commercial software solution to 3D scanning**. Graphical Models, 2005, 67 (6): 476-495.

[4] Bouguet, J.-Y**. Camera Calibration Toolbox for Matl**ab.

<http://www.vision.caltech.edu/bouguetj/calib_doc/index.html>

[5] Woebbecke, D., Meyer, G., Von Bargen, K., et al. **Color indices for weed identification under various soil, residue, and lighting conditions**. Transactions of the ASAE, 1995, 38 (1): 259-269.

[6] Meyer, G., Mehta, T., Kocher, M., et al. **Textural imaging and discriminant analysis for distinguishingweeds for spot spraying**. Transactions of the ASAE, 1998, 41 (4): 1189.

[7] Jiang, N., Yang, W., Duan, L., et al. **A nondestructive method for estimating the total green leaf area of individual rice plants using multi-angle color images**. Journal of Innovative Optical Health Sciences, 2015, 08 (02): 1550002.

[8] Neto, J. C., Meyer, G. E., Jones, D. D. **Individual leaf extractions from young canopy images using Gustafson–Kessel clustering and a genetic algorithm**. Computers and Electronics in Agriculture, 2006, 51 (1-2): 66-85.

[9] Geiger, A., Roser, M., Urtasun, R. **Efficient large-scale stereo matching**. In Computer Vision–ACCV 2010, Springer: 2010; 25-38.

[10] Cochran S D, Medioni G. **3-D surface description from binocular stereo**. IEEE Transactions on Pattern Analysis & Machine Intelligence, 1992, 14(10):981-994.

[11] Fua P. **A parallel stereo algorithm that produces dense depth maps and preserves image features** . Machine Vision & Applications, 1993, 6(1):35-49.

[12] C. Barber, D. Dobkin, H. Huhdanpaa, **The quickhull algorithm for convex hulls**, ACM Transactions on Mathematical Software (TOMS) 22 (1996) 469–483.

[13] Edelsbrunner H, Seidel R. **Voronoi diagrams and arrangements**[J]. Discrete & Computational Geometry, 1986, 1(1):25-44.
