# Supplementary material for: A high-throughput stereo-imaging system for quantifying rape leaf traits during the seedling stage
Source: Plant Methods. 2017 Jan 31;13:7. doi: 10.1186/s13007-017-0157-7 (PMC5282657; doi:10.1186/s13007-017-0157-7)
Supplement: Supplementary file 13 — Additional file 13. Supplementary document for individual leaf traits. [file 13007_2017_157_MOESM13_ESM.doc]

**Individual leaf traits**

The main content of this part is to describe the specific image processing steps for individual leaf extraction. This part provides a nondestructive method to analysis individual leaf traits and it is an independent mode different from the canopy reconstruction. Here, we would expect to extract individual leaf traits just from one top-view image taken by stereo-imaging system. A user interaction process is applied to extract individual leaf with more complete and accurate. The detailed operating steps are show in video (Additional File 5).

**Choose individual leaf**

Here, a user interaction process is applied to extract individual leaf. The user needs to click the left mouse button and drag it to choose a rectangular box. In this rectangular box, the individual leaf must be typical and representative, which means that the chosen leaf should have stable phenotypic traits and uniform size. When the user is not satisfied the result of choosen leaf region, clicking any key to return or press “Enter” for confirmation. All individual leaf images are saved in PNG format for subsequent analysis

**Rotate rectangular image**

Usually, the selected leaf has a long petiole part, the existence of which will seriously impact the analysis of blade traits. In order to facilitate the petiole removal process, here, the purpose of rotating rectangular image is to make petiole’s direction downward.

Firstly, the user needs to mark two points on the petiole. The first point represents the intersective point of petiole and blade. Also, the first point's y coordinate on the rotated image is the end position of petiole searching. And, the vector from first point to second point decides the rotate angle of image rotating. The detailed computational expressions are as follows 1-2:

$X=x_{0}\times\cos\theta+y_{0}\times\sin\theta-0.5\times W\times\cos\theta-0.5\times H\times\sin\theta+0.5\times W_{n}$ (1)


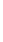
 (2)

Where, *(x_0_,y_0_)* are the first point’s coordinate of unrotated image; *(X,Y)* are the first point’s coordinate of rotated image; *θ* is the counterclockwise rotation angle; *W* is the width of the unrotated image, *H* is the height of the unrotated image; *W_n_* is the width of the rotated image, *H_n_* is the height of the rotated image. The vector from first point to second point is to determine the direction of rotation.

**Individual leaf segmentation**

The main content of this part is to segment green region from rotated rectangular image. Here, the segmentation algorithm adopts the same method in Additional File 9. The excess green vegetation index (*ExG*) [1] and excess red vegetation index (*ExR*) [2] are used. The detailted expressions are as follows 3-6:

$R=\frac{r}{(r+g+b)}\text{ }G=\frac{g}{(r+g+b)}\text{ }B=\frac{b}{(r+g+b)}$ (3)

$ExG=2\times G-R-B\text{ > Threshold\_G ? 1 ,0}$ (4)

$ExR=1.4R-G>\text{ Threshold\_R ? 1 , 0}$ (5)

$bw=ExG-ExR$ (6)

Where, *r*, *g* and *b* indicate the pixel values of the red, green and blue channels, respectively. The result of segmentation is shown in Figure 11d.

**Remove the petiole region**

Rape leaf in seedling stage can divide into two parts: petiole and blade. The existence of the petiole will impact analysis of blade phenotypic traits. However, for the non-destructive imaging acquiring, the selected rectangular images will inevitably exist petiole. So, the next step is to remove petiole. The difference between blade and petiole in color and texture is tiny. But from the view of shape, petiole region is more slender than blade. After above steps, the direction of petiole is downward. Therefore, we only need to search the binary leaf image from bottom to top to remove foreground pixels, which horizontal width less than a specified threshold area. Here, the area threshold is set to 25, which is an appropriate value determined by lots of preliminary experiments.

**Connected components screening and small holes fill**

The situation that other partial leaf region might be choosen in the rectangular region was always happened. So, the main content of this part is to keep only one foreground targret component. After the observation, the target individual leaf region occupied the middle of image, while the impurities were around the edge of image and smaller than target region. Considering this situation, a specified area threshold was developed to screen connectd component. When the region was smaller than the area threshold, we would exclude them. Usually, the target leaf region had the largest area. So, only thing we need to do is to keep the largest connected component as the target individual leaf (Figure 11e).

In addition, because of the binarization segmentation error, small holes (the red box in Figure 11e) might appear on the target area. These holes usually have small number of pixels comparing with other real holes. An area threshold (the initial threshold is set to 100) is used to fill small area of holes. The result of segmentation is shown in Figure 11f.

**Phenotypic parameters extraction**

There are 19 individual leaf shape traits, including 11 scale-invariant traits, 3 inner cavity related traits, and 5 margin related traits were extracted. The minimum circumscribed rectangle was the common image analysis method in morphological measurement. To get the minimum circumscribed rectangle, the image should search from left to right and from top to bottom, respectively. For the first search, the left-most foreground pixel in single connected component was found as the left border of circumscribed rectangle. The width of the circumscribed rectangle also can be obtained by calculating the horizontal distance of the left-most pixel to right-most pixel. The height of circumscribed rectangle also can be obtained in the same way. Morever, the convex hull [5] is anothor important method to represent the traits of target objects. The definition of convex hull is the minimum convex polygon region, which contains all pixels of target objects. The third image processing skill is elipse fitting [6]. For the rape leaf binary image, we should firstly extract the coordinates of the outline pixels by using "canny" algorithm. Then, the least squares algorithm was choosen as the main algorithm to calculate external elliptical expression.

For the serrations and indents, the adjacent two serration points contains a section of outer boundary pixels, which could be storaged in the form of linked list. It means that all the pixels between these two adjacent connected points can be extacted separately to calculate the euclidean distance to the corresponding conves hull line. Computational expressions are as follows:

$P(\mathrm{i})=\{(\mathrm{x}_{i},\mathrm{y}_{i})|\mathrm{i}\in[1,n]\}$ (7)

$A\times x+B\times y+C=0$ (8)

$D(\mathrm{i})=\frac{|\mathrm{A}\times\mathrm{x}_{i}+\mathrm{B}\times y_{i}+\mathrm{C}|}{\sqrt{A^{2}+B^{2}}}$ (9)

Where, $p(\mathrm{i})$are the pixels between two adjacent points;$A\times x+B\times y+C=0$are the linear equation of two adjacent serration points. $D(\mathrm{i})$ is the euclidean distance from the pixels between two adjacent serration points to corresponding conves hull line

For each section of outer boundary pixels, the distance to corresponding conves hull line may be regarded as a continuous curve function. The local maximum values expressed the indents number. Also, in the same section of outer boundary pixels, the number of local maximum values always not just one. In addition, not all the local maximum points are effective indents. So a threshold was choosen to be a predefined distance value. Only the value of local maximum greater than this threshold, the indent point would be saved. The effectiveness of indents is calculated by using the following equation 10. When the ratio is bigger than 0.3, we consider the indents are effective indents.

$iseffective=\frac{depth}{\min(height,width)/2 }$ (10)

Where, *height* and *width* represent the number of minimum circumscribed box’s rows and cols, respectively. Also, *depth* represents the distance from indent point to corresponding convex hull straight line

**References**

[1] Woebbecke, D., Meyer, G., Von Bargen, K., et al. **Color indices for weed identification under various soil, residue, and lighting conditions**. Transactions of the ASAE, 1995, 38 (1): 259-269.

[2] Meyer, G., Mehta, T., Kocher, M., et al. **Textural imaging and discriminant analysis for distinguishingweeds for spot spraying**. Transactions of the ASAE, 1998, 41 (4): 1189.

[3] Jiang, N., Yang, W., Duan, L., et al. **A nondestructive method for estimating the total green leaf area of individual rice plants using multi-angle color images**. Journal of Innovative Optical Health Sciences, 2015, 08 (02): 1550002.

[4] Neto, J. C., Meyer, G. E., Jones, D. D. **Individual leaf extractions from young canopy images using Gustafson–Kessel clustering and a genetic algorithm**. Computers and Electronics in Agriculture, 2006, 51 (1-2): 66-85.

[5] Jarvis, R. A**. On the identification of the convex hull of a finite set of points in the pla**ne. Information Processing Letters, 1973, 2 (1): 18-21.

[6] Gander, W., Golub, G. H., Strebel, R. **Least-squares fitting of circles and ellipses**. BIT Numerical Mathematics, 1994, 34 (4): 558-578.
