# Supplementary material for: A high-throughput stereo-imaging system for quantifying rape leaf traits during the seedling stage
Source: Plant Methods. 2017 Jan 31;13:7. doi: 10.1186/s13007-017-0157-7 (PMC5282657; doi:10.1186/s13007-017-0157-7)
Supplement: Supplementary file 14 — Additional file 14. Supplementary document for three classification methods. [file 13007_2017_157_MOESM14_ESM.doc]

**Three classification methods**

The main content of this part is to describe the theorys and specific implementation for three calssification methods, including stepwise discriminant analysis (SDA), support Vector Machine (SVM) and rondom forest (RF).

**Stepwise discriminant analysis**

The specific operating approaches are as follows: All variables are selected as the input variables of the algorithm. Then, the SDA algorithm will select a variable that has the most significant discriminant ability. Next, the selecting for second variable based on the first one, which indicates that combining the first and second variables will have the most significant discriminant ability. By that analogy, the third variable will be selected. Because of the mutual relationship between different variables, the previous variable may lose significant discriminant ability after inputting the new variable. Then, we will inspect the discriminant ability of all previous selected variables to find the disabled variables, remove them, and go on to find new variables until no significant variables can be removed.


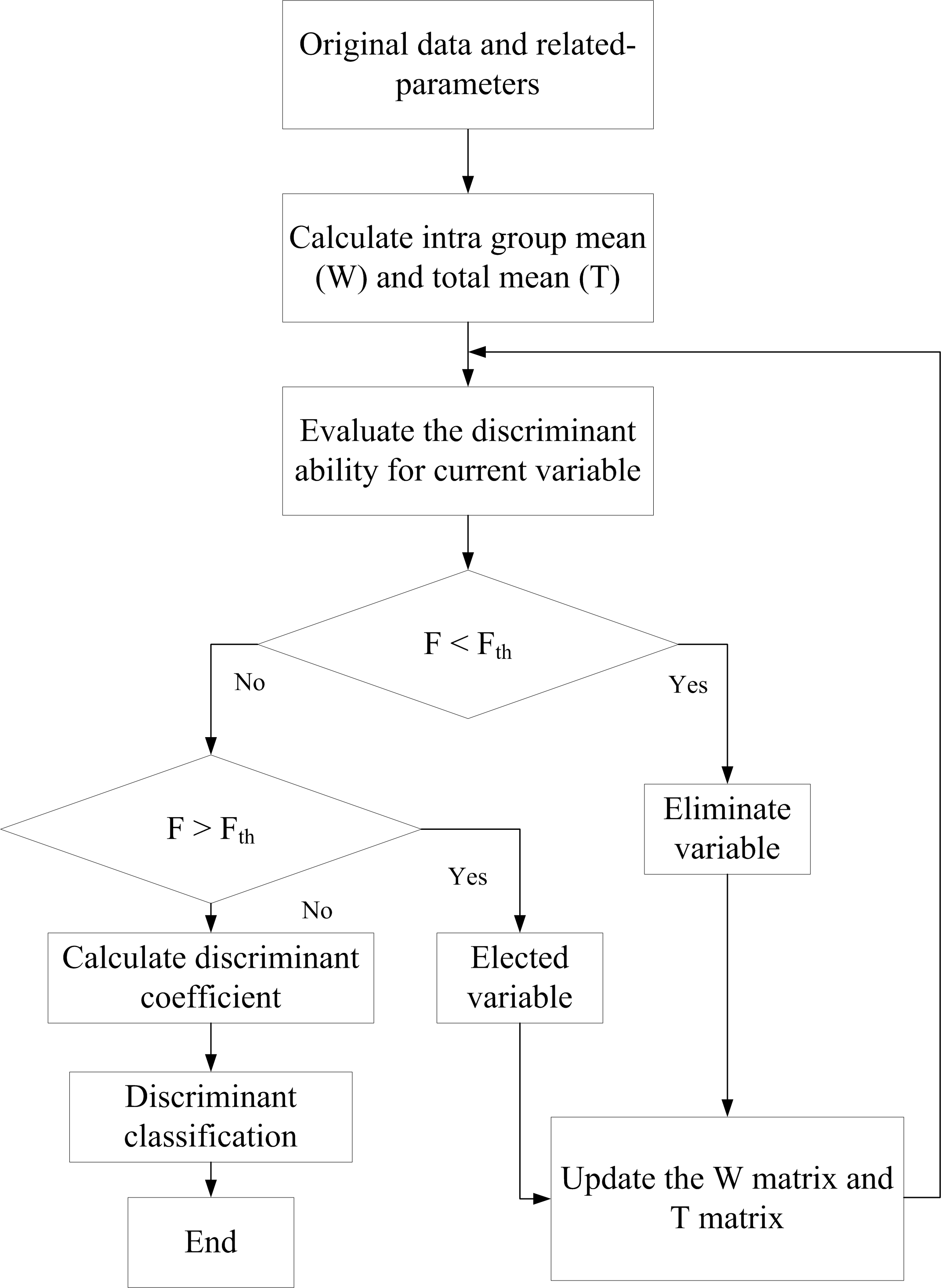


Figure 1 Stepwise discriminant analysis flow

The detailted algorithm process is shown in Figure 1. In this study, stepwise discriminant analysis training was achieved using (SPSS v.22 software), which is a proven technique for meaningfully classifying different shapes [1]. 402 samples for training are used to build SDA linear model. Finally, the testing group (399 samples) is classified using above model.

**Support Vector Machine**

The support Vector Machine (SVM) is a common supervised learning algorithm that has been shown to provide state-of-the-art performance in many classiﬁcation problems. In this study, the LIBSVM-matlab toolkit [2] was used here to conduct SVM model. All the 801 samples with three different leaf shapes were randomly divided into two groups (402 samples for training and 399 samples for testing). Firstly, we should limit all data into a certain range. The purpose for data normalisation is to ensure the convergence of the SVM. At the same time, the data normalisation improves the accuracy of classification. The detailed normalized mapping is shown in Equation 1:

$f:\mathrm{x}\to\mathrm{y}=\frac{x-x_{\min}}{x_{\max}-x_{\min}}$ (1)

The Normalized effect is to structure the data into interval from 0 to 1. The implementation method is to use the mapminmax function in Matlab2013. Next, we can train the model by using training samples. Here, the kernel function is generated to use polynomials and the kernel parameter is set to 1.5. Also, the penality parameter is set to 2. The genetic algorithm (GA) was used to choose the best value of kernel parameter and penality parameter. In the field of mathematical optimization, the GA is a search heuristic that mimics the process of natural selection. This heuristic is routinely used to generate useful solutions to optimization and search problems. Finally, the front model will be used to deal with testing samples. The detailed algorithm code refers to Additional File 14.

**Rondom forest**

The random forest (RF) classifier is a combination of multiple decision trees. In addition to constructing each tree using a different bootstrapped sample of data, random forests change how the trees are constructed. In standard trees, each node is split using the best split among all variables and the best among a subset of predictors randomly chosen at that node. The detailed random forests algorithm (for classiﬁcation) is as follows:

1. Draw ntree bootstrap samples from the original data.

2. For each of the bootstrap samples, grow an unpruned classiﬁcation tree, with the following modiﬁcation: at each node, rather than choosing the best split among all predictors, randomly sample mtry of the predictors and choose the best split from among those variables. (Bagging can be thought of as the special case of random forests obtained when mtry = p, the number of predictors.)

3. Predict new data by aggregating the predictions of the ntree trees (i.e., majority votes for classiﬁcation).

In this study, the open source randomforest-matlab toolkit [3] was adopted to build random forest classifier. Abhishek Jaiantilal, of the University of Colorado, Boulder, is the primary developer. Here, the number of decision trees in my random forest is set to 1000 and the other parameters adopt the default value. 801 samples were randomly selected and divided into two groups: 402 samples comprising the training group and 399 samples for testing group. When the test samples enter into the random forest, every decision treess will independently classify the category it belongs to.

The detailed algorithm code refers to Additional File 15.

**References**

[1] Petalas, C., Anagnostopoulos, K. **Application of Stepwise Discriminant Analysis for the Identification of Salinity Sources of Groundwater**. Water Resources Management, 2006, 20 (5): 681-700.

[2] Chang, C.-C., Lin, C.-J. **LIBSVM: a library for support vector machines**. ACM Transactions on Intelligent Systems and Technology (TIST), 2011, 2 (3): 27.

[3] Liaw A, Wiener M. **Classification and Regression by RandomForest**. R News, 2001, 23(23).
